# Prevalence of polypharmacy and potentially inappropriate prescribing in older adults with a prognosis of 6 months or less: a systematic review and meta-analysis

**DOI:** 10.1093/ageing/afag326

**Published:** 2026-11-13

**Authors:** Xin Er Yaw, Yen Jun Wong, Wing Loong Cheong, J Simon Bell, Ednin Hamzah, Shaun Wen Huey Lee

**Affiliations:** School of Pharmacy, Monash University Malaysia, Jalan Lagoon Selatan, Bandar Sunway, 47500 Subang Jaya, Selangor, Malaysia; School of Pharmacy, Monash University Malaysia, Jalan Lagoon Selatan, Bandar Sunway, 47500 Subang Jaya, Selangor, Malaysia; School of Pharmacy, Monash University Malaysia, Jalan Lagoon Selatan, Bandar Sunway, 47500 Subang Jaya, Selangor, Malaysia; Monash University Faculty of Pharmacy and Pharmaceutical Sciences, Centre for Medicine Use and Safety, Parkville, VIC 3052, Australia; Hospis Malaysia, Cheras, Kuala Lumpur, Malaysia; School of Pharmacy, Monash University Malaysia, Jalan Lagoon Selatan, Bandar Sunway, 47500 Subang Jaya, Selangor, Malaysia

**Keywords:** polypharmacy, potentially inappropriate medications (PIM), end of life, palliative care, deprescribing, systematic review, older people

## Abstract

**Background:**

Polypharmacy and potentially inappropriate medications (PIMs) are common in older adults, but the prevalence in those with a prognosis of 6 months or less is poorly characterised. We estimated the prevalence of polypharmacy, PIMs and potential prescribing omissions (PPOs) in older adults approaching end of life.

**Methods:**

We systematically searched six databases from inception to 30 April 2026, for studies reporting polypharmacy (≥5 medications) or PIMs in adults aged ≥65 years with life-limiting illnesses (≤6-month prognosis). Two reviewers independently screened records, extracted data and assessed bias using Joanna Briggs Institute or Newcastle-Ottawa tools. Pooled prevalence used random-effects models. Heterogeneity was explored by setting, region and assessment tool. The review was registered with PROSPERO (CRD42023454636).

**Findings:**

Eighteen studies (499 853 participants, 52.3% female) were included in this review. Pooled prevalence of polypharmacy was 76% (95% CI 65%–87%) with excessive polypharmacy (≥10 medications) at 34% (9%–58%). PIM prevalence was high at 54% (40%–68%), and remained above 50% until the final week of life, with preventive medications (statins, antithrombotics, proton pump inhibitors) often continued until death. Substantial heterogeneity was present in all pooled analyses (*I*^2^ > 99%), highlighting considerable variability across studies. Only two studies assessed PPOs 30% lacked prophylactic laxatives with opioids while 76.6% lacked strong opioids for moderate–severe cancer pain.

**Interpretation:**

Polypharmacy and PIMs use are highly prevalent among people with limited life prognosis, suggesting that evidence-based deprescribing protocols are needed for this vulnerable population. Nevertheless, this needs to be taken in light of the substantial heterogeneity of PIM definitions, underscoring the need for standardised PIM assessment in this population.

## Key points

- Three-quarters of older adults with a prognosis of 6 months or less have polypharmacy (≥5 medications), and half use potentially inappropriate medications (PIMs).
- PIM prevalence remains above 50% until the final week of life, driven largely by continued use of preventive medications (statins, antithrombotics, proton pump inhibitors) whose time-to-benefit exceeds remaining life expectancy.
- PIM prevalence varies substantially by assessment tool (ranging from 23% with STOPPFrail V1 to 91% with general geriatric criteria), highlighting a ‘measurement paradox’ that complicates cross-study comparisons.
- Proactive deprescribing at key transitions (e.g. hospice enrolment, change in goals of care) and pharmacist-led, trigger-based medication reviews (e.g. ≥5 medications) can reduce PIM burden and align prescribing with palliative goals.
- Evidence on potential prescribing omissions (PPOs) is critically scarce, and prospective research is urgently needed to address undertreatment of symptoms (e.g. pain, constipation) at the end of life.

## Introduction

Medication management in older adults approaching end-of-life is complex. For many of these older adults, polypharmacy (or the use of ≥5 medications) are common due to multimorbidity. Studies have shown that approximately one in five people with life-limiting illnesses receive eight or more medications [1], many which are used as preventive treatments (statins, bisphosphonates, aspirin) [2, 3]. As the aim of palliative care is to optimise quality of life for individuals with life-limiting illnesses, the use of such preventive treatments are often classified as potentially inappropriate medications (PIMs) as care goals transitions from curative to comfort-focused management and symptom control [2–4].

Therefore, preventive medications with prolonged time-to-benefit offer minimal returns for patients with limited prognosis as it increases treatment burden and adverse event risk [5]. For example, primary prevention with statins requires approximately 2.5 years to prevent one event per 100 patients [6]. Similarly, PIM use in hospitalised older adults is associated with a 91% increase in adverse drug event-related admissions and a 60% increase in functional decline [7]. These harms are magnified especially in end-of-life populations due to the diminished physiological reserve. Conversely, under-prescribing especially for symptom relief can result in suffering [8].

As such, regular medication review is integral for this population to ensure that prescribing remains aligned with goals of care. The process resembles medication reviews in older adults generally, but carries two additional emphases, deprescribing of medicines where time to benefit exceed remaining life expectancy and anticipatory prescribing for the last days of life.

However, targeting this process remains difficult, as the prevalence of PIMs and PPO remains poorly characterised in this population [9]. This systematic review and meta-analysis aimed to estimate the prevalence of polypharmacy, PIMs and PPOs in adults aged ≥65 years with a prognosis of six months or less.

## Methodology

This review followed PRISMA 2020 and was registered with PROSPERO (CRD42023454636) [10, 11]. See Supplementary Appendix 1 for the completed PRISMA checklist.

### Study registration

This review was prospectively registered on PROSPERO (CRD42023454636). Deviations from the registered protocol include: (i) expansion of the search timeframe from 31 July 2023, to 30 April 2026, to capture more recent evidence; (ii) change in age eligibility from ≥60 to ≥65 years to align with standard geriatric definitions; (iii) addition of medication burden as a secondary exploratory outcome identified during data extraction, as multiple included studies reported mean/median medication counts; (iv) removal of impact outcomes (symptom management, quality of life, functional impairment, adverse drug events, medication costs, patient satisfaction) due to insufficient data across studies and (v) the ‘PIM measurement paradox’ was an emergent finding observed during data extraction and analysis, rather than a pre-specified objective. All deviations were made prior to data extraction and are justified in the methods.

### Search strategy

We searched PubMed, Embase, CINAHL Plus, PsycINFO, Web of Science and Scopus from inception to 30 April 2026, using MeSH headings and keywords for prevalence, older adults, life-limiting illness, end-of-life care, polypharmacy, PIMs and PPOs (see Supplementary Appendix 2 for the full details). We did not search websites, organisations or trial registers. The concepts ‘life-limiting illness’ and ‘end-of-life care’ were combined using AND to prioritise specificity over sensitivity, given the broad scope of the review. To compensate for potential missed records, we hand-searched reference lists of included studies and relevant reviews. Reference lists of eligible articles were also searched. We did not conduct a grey literature search.

### Eligibility criteria

We included studies reporting polypharmacy (defined as the use of ≥5 medications) or inappropriate prescribing in adults aged ≥65 years during the last 6 months of life. Studies with participants <65 years were included if data for those ≥65 years could be extracted. All PIM assessment tools were considered eligible, irrespective if these were initially developed for the general geriatric population (e.g. Beers, STOPP/START, Laroche) or those with limited prognosis or palliative care needs (e.g. STOPPFrail, OncPal, Morin criteria). This inclusive approach was taken to examine the variation in prevalence estimates attributable to the choice of assessment tool. Findings were referred to as the ‘PIM measurement paradox’ in this review. All studies irrespective of study design were included, if they reported baseline (pre-intervention) prevalence data [12]. In such cases, only baseline estimates were extracted for meta-analysis. For excessive polypharmacy, we used author-defined thresholds (most commonly defined as ≥10 medications, as the ≥9 medication used for nursing home studies were not reported) [13]. We excluded studies that did not examine pharmacotherapy, editorials, commentaries, letters, conference abstracts and non-English studies.

### Study selection, data extraction and quality appraisal

Records were de-duplicated in EndNote and screened in Rayyan. Titles and abstracts were screened independently by two reviewer teams. Full-text articles were retrieved and screened independently by two reviewers, with disagreements resolved by consensus with a third reviewer. Data were extracted using a standardised form by one reviewer and independently checked by a second reviewer. During our review, medication burden was included as a secondary exploratory outcome while study outcomes (e.g. symptom management, quality of life, functional impairment, adverse drug events, medication costs, patient satisfaction) were excluded due to insufficient data. Authors were contacted for missing data. Risk of bias was assessed independently by two reviewers, with disagreements resolved by consensus with a third reviewer, using the JBI Checklist for cross-sectional studies and the Newcastle-Ottawa Scale for cohort studies [14, 15].

### Data synthesis

We narratively described all studies and performed meta-analyses of prevalence for polypharmacy, excessive polypharmacy and PIMs using the DerSimonian and Laird random-effects model, with heterogeneity measured by *I*^2^ [16]. Outcomes were reported based on the most specific to the end-of-life/palliative care context using the following hierarchy: STOPPFrail V2 > STOPPFrail V1 > other palliative-specific tools > general geriatric criteria. A detailed overview of the different PIM assessment tool is summarised in Table 1. In the event multiple tools were used in a study, the hierarchy rule above was followed in our reporting. For studies that did not use a formal PIM tool but operationalised PIMs through specific medication classes (e.g. Mack *et al*. [28] on statins; Springer *et al*. [29] on aspirin), we extracted the prevalence of those specific medication classes as PIMs, as they were explicitly framed as medications of questionable benefit in the end-of-life context.

**Table 1:** Comparative summary of different PIM assessment tools used in included studies.

**Clinical tool**	**Intended target population of tool**	**NO. OF CRITERIA**	**PIM representative examples**
**General geriatric tool**			
2019 Beers Criteria [17]	General older adults aged ≥65 years	30 individual medication criteria; 16 drug-disease/syndrome criteria	Benzodiazepines, antipsychotics, NSAIDs, sulfonylureas, skeletal muscle relaxants, digoxin, aspirin for primary prevention, SNRIs, SSRIs, TCAs, dabigatran, rivaroxaban.
Laroche criteria [18]	General older adults aged ≥75 years. French adaptation of Beers criteria	34 (29 general, 5 disease-specific)	Ticlopidine, glipizide, glibenclamide, amitriptyline, imipramine, doxepin (>6 mg), indomethacin, phenylbutazone, nitrofurantoin, short-acting nifedipine, long-acting benzodiazepines, anticholinergic drugs (oxybutynin, atropine), NSAIDs in heart failure/renal failure
INTERCheck [19]	Older adults with multimorbidity and complex polytherapy	Integrated software where multiple tools (Beers Criteria, START/STOPP Criteria, STOPPFrail Criteria, ACB Scale, DDI database, renal dose adjustment, GerontoNet ADR Risk Score) are integrated	Derived from Beers, START/STOPP and STOPPFrail criteria, e.g. benzodiazepines, antipsychotics, NSAIDs, statins, aspirin, digoxin, duplicate drug classes, drugs with interactions
STOPP criteria (2015) [20]	General older population aged ≥65 years	80 criteria (part of 114 STOPP/START criteria)	Benzodiazepines, antipsychotics, NSAIDs, duplicate drug classes, drugs with drug–drug interactions, PPIs (full dose ≥8 weeks), drugs that adversely affect fallers, cardiovascular drugs with adverse interactions, CNS-active drugs with adverse effects
**Palliative/end-of-life-specific tools**			
Holmes criteria [21]	Advanced dementia (FAST 6E-7C) nearing end of life	69 criteria achieved consensus, 4-tier classification: Never appropriate, Rarely appropriate, Sometimes appropriate and Always appropriate	Never appropriate: Lipid-lowering medications (statins), NMDA receptor antagonists (memantine), antiplatelet agents (excluding aspirin), acetylcholinesterase inhibitors (AChEIs), cytotoxic chemotherapy, antioestrogens, sex hormones, immunomodulators Rarely appropriate: α-blockers (excluding tamsulosin), digoxin, bisphosphonates, clonidine, antiarrhythmics, hydralazine, heparin and LMWH, warfarin, antiandrogens, appetite stimulants, bladder relaxants, tamsulosin, antispasmodics, mineralocorticoids
Morin criteria [22]	Older adults aged ≥75 years with life expectancy ≤3 months	52 drug classes (14 adequate, 28 questionable, 10 inadequate for continuation; 10 adequate, 23 questionable, 23 inadequate for initiation)	Inadequate: Statins, bisphosphonates, antidementia drugs (AChEIs, memantine), vitamin D, calcium supplements, antihypertensives (excluding α-blockers), lipid-modifying agents, warfarin/NOACs, antineoplastic drugs
OncPal [23]	Palliative cancer patients with life expectancy <6 months	8 medication classes	Antihypertensives (amlodipine, ramipril), lipid-lowering agents (statins), anticoagulants/antiplatelets (warfarin, aspirin, clopidogrel), oral hypoglycaemics (metformin), PPIs (omeprazole), CAMs, vitamins/mineral supplements, bisphosphonates (alendronate)
PIP-CPC [24]	Older adults aged ≥65 years with cancer receiving palliative care and life expectancy <1 year	24 criteria (symptom-specific) (anorexia-cachexia 1; anxiety 2; constipation 5; delirium 1; depression 3; diarrhoea 1; dyspnoea 1; fatigue 2; insomnia 2; nausea/vomiting 2; pain 3; duplicate drug classes 1)	Opioids without laxatives; benzodiazepines >4 weeks; TCAs as first-line for depression; hyoscine as first-line for nausea; NSAID + corticosteroid without PPI; absence of short-acting opioid for breakthrough pain; duplicate drug classes
STOPPFrail V1 [25]	Frail older adults aged ≥65 years with: End-stage pathology, poor 1-year prognosis, severe functional/cognitive impairment, symptom control priority	27 criteria (General 2, CVS 2, Coagulation 1, CNS 2, GI 3, Respiratory 2, MSK 5, Urogenital 3, Endocrine 4, Miscellaneous 3)	Statins, aspirin (primary prevention), PPIs (≥8 weeks), neuroleptics (>12 weeks), bisphosphonates, calcium supplements, memantine, theophylline, oral NSAIDs (≥2 months), oral steroids (≥2 months), ACE inhibitors/ARBs (diabetic nephropathy only), multivitamins
STOPPFrail V2 [26]	Older people approaching end of life: ADL dependency and/or severe chronic disease and/or terminal illness; severe irreversible frailty	25 criteria (General 3, Cardiology 3, Coagulation 2, CNS 2, GI 2, Respiratory 2, MSK 5, Urogenital 2, Endocrine 1, Miscellaneous 3)	Statins; antihypertensives (if SBP <130 mmHg); anti-anginals; antiplatelets (primary prevention); aspirin (AF); neuroleptics (>12 weeks); memantine; PPIs (≥8 weeks); theophylline; calcium; vitamin D; bisphosphonates; oral NSAIDs (≥2 months); oral steroids (≥2 months); anti-diabetics (de-intensify); multivitamins; folic acid
Todd criteria [27]	Patients with life-limiting illness near the end of life	Conceptual framework (no fixed number): Considers life expectancy, time until benefit, goals of care, treatment targets	Lipid-lowering agents (statins), antihypertensives, antidiabetic medications, antiplatelet agents, vitamins/minerals, antiulcer medication

Medication burden outcomes (mean and median number of medications) were narratively reported as a secondary exploratory outcome due to the lack of standardised data across studies. Results were stratified by study setting, region and assessment tool. Sensitivity analyses excluding studies with moderate/high risk of bias, small sample size (<5000) or < 10% female were also performed. Publication bias was assessed using Doi plots and LFK index [30]. All analyses used Stata 19.5 (StataCorp, College Station, Texas, USA).

## Results

### Search results

Of the 18 887 identified studies, 204 underwent full-text screening and 18 met inclusion criteria (Figure 1). Common reasons for exclusion included: included adults aged <65 years (*n* = 99), participants had >6 months or unclear prognosis (*n* = 51), editorials/abstracts (*n* = 27), non-English (*n* = 2), not relevant to polypharmacy/PIM/PPO (*n* = 6) or excluded end-of-life patients (*n* = 1) (see Supplementary Appendix 3 for the full list of excluded studies).

**Figure 1:**
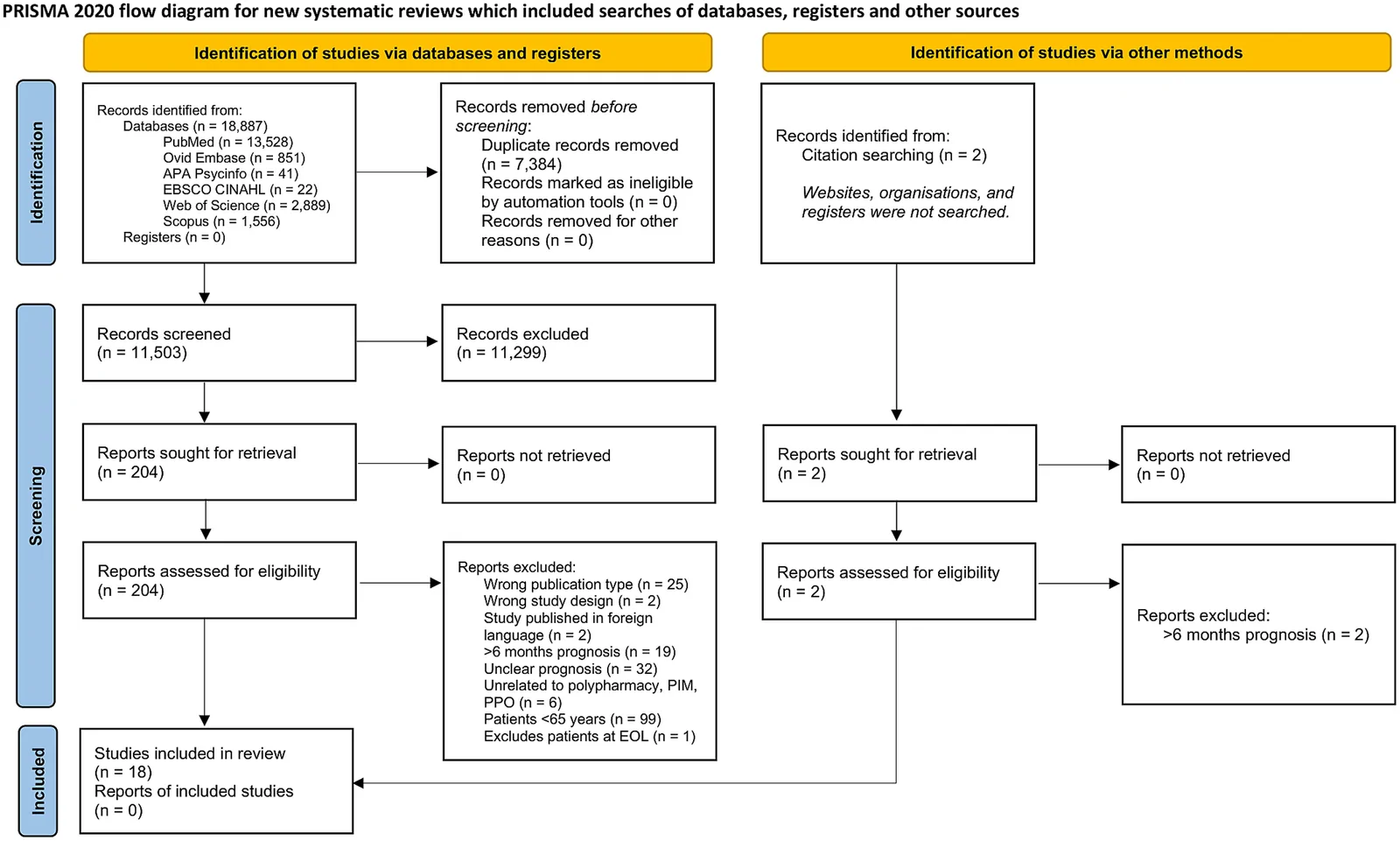
PRISMA flowchart.

The 18 studies included 499 853 participants with a mean/median age of 69–88.8 years and 52.3% were female. Eleven studies were from Europe, four from Asia and three from North America. These studies were conducted in hospitals [12, 31, 32], hospices [33–35], nursing homes [28, 29, 36–40], community/outpatient [41] or were national registries [42–45]. The characteristics of included studies are summarised in Table 2 and Supplementary Appendix 4. Half of the studies were rated as high quality (*n* = 9) with the remaining nine rated as moderate quality (see Supplementary Appendix 5).

**Table 2:** Study characteristics across included articles.

**Author, year**	**Study design, Setting**	**Country (Region)**	**Sample size (n)**	**Age (Years)**	**Female (%)**	**Study period**	**Life expectancy**	**Life-limiting illnesses (%)**	**Other Comorbidities**	**PIP clinical tool**
Alwidyan, 2024	Retrospective cohort, Hospices	Northern Ireland (Europe)	106	65–69 years (21.7%) 70–74 years (27.4%) 75–79 years (23.6%) 80–84 years (16%) 85–89 years (5.7%) ≥90 years (5.7%)	63.2	October 2019 to January 2021	Last 2 weeks	Cancer (92.5) COPD (1.9) Pneumonia (0.94) Parkinson’s disease (0.94) Pulmonary fibrosis (0.94) Respiratory failure (0.94) End stage renal disease (0.94) Motor neuron disease (0.94)	Mean no. of chronic conditions per patient: 3.9 (1.6)	STOPPFrail, Morin criteria
Anlay, 2025	Retrospective cohort, Register based cohort	Belgium (Europe)	244,865	≥85 years (67.7%)	65.72	January 2014 to December 2019	Last 4 months	Dementia and cancer identified via cause of death; specific percentages not provided.	Not reported	STOPPFrail V1, Bruyere Deprescribing.org drug-specific guidelines (for PPIs and antipsychotics)
Chen, 2010	Retrospective cohort, Nursing home	Taiwan (Asia)	31	Mean: 83.16 (7.59)	48	N/A	Last 6 months	Lung disease/infection (51.6) Heart disease (16.1) Cancer (9.7) Sepsis (9.7) Stroke (6.5)	N/A	N/A
Ham, 2022	Retrospective cohort, Register based cohort	Netherlands (Europe)	7864	Median: 74 (9)	33	2009 to 2014	Last 1 month	Lung cancer (100)	Cardiovascular diseases (52%) T2DM (18%) Kidney disease (3%) Pulmonary disease (32%)	OncPal
Hattori, 2025	Retrospective cohort, Outpatient/Community healthcare setting	Japan (Asia)	8463	75–79 years (24.4%) 80–84 years (30.2%) 85–89 years (26.2%) 90–94 years (14.7%), ≥ 95 years (4.5%)	46.8	October 2017 to September 2019	Last 6 months	Advanced-stage cancers: Lung (21.1) Colorectal (19.2) Gastric (16.5) Pancreatic (10.5) Prostate (10.2) Hepatobiliary (8.0) Breast (5.0) Female genital organs (4.0) Kidney/ureter (4.1) Bladder (5.1) Haematological (6.0)	Heart failure (44.7) T2DM (43.9) IHD (29.1) Dementia (26.3) Asthma/COPD (25.9) Stroke (18.5) Depression (16.7) Parkinson’s disease (3.0)	Morin et al., OncPal, Hattori et al., Tjia et al., STOPPFrail v2
Lee, 2025	Retrospective observational, Hospice	South Korea (Asia)	78	Mean: 81.3 (6.5)	42.3	November 2022–2023	Last 6 months	Cancer (100): Lung (23.1) Gastric (7.7) Colorectal (10.3) Hepatic (12.8) Pancreatic (14.1) Other (33.3)	Hypertension (62.8) Diabetes (35.9) Dyslipidaemia (35.9) ECOG-PS1 (0) ECOG-PS2 (19.2) ECOG-PS3 (50.0) ECOG-PS4 (30.8) Cancer pain (59.0)	PIM: STOPPFrail V2, PPO: Defined as absence of strong opioids in patients with moderate to severe cancer pain based on WHO
Mack, 2020	Cross-sectional, Nursing home	United States (North America)	13,241	Range: 65–75 years (15.06%) > 75 years (84.94%)	70.7	April to September 2016	Last 6 months	Cancer (4.8) COPD/Lung disease (32.7) Dementia (58.2) ESRD (3.7) Heart failure (30.5) Hip fracture (3.6) Liver disease (0.9) Neurologic (e.g. ALS) (0.3) CAD (24.3) Peripheral vascular/artery disease (13.6) Stroke (14.4)	T2DM (20.9%) Dyslipidaemia (47.8%) HTN (81.5%)	N/A
Matlow, 2017	Cross-sectional, Nursing home	Canada (North America)	9298	Mean: 86.4 (6.7)	64.9	1 June 2010 to 31 March 2013	Last 4 months Last week	Advanced dementia (100)	HTN(61.6%) Stroke (29.9%) T2DM (29.1%)	Holmes criteria
Morin, 2019	Retrospective cohort, Register based cohort	Sweden (Europe)	58,415	Mean: 87.0 (6.3)	55.9	1 January to 31 December 2015	Last 3 months	Cancer (28) Organ failure (40) Prolonged dwindling (32)	Mean no. of chronic diseases: 6.6 (3.2) Chronic diseases: 0–1: 2.9% 2–3: 13.8% 4–5: 23.1% >6: 60.2%	Morin criteria
Murphy, 2022	Retrospective cohort, Inpatient palliative care	Ireland (Europe)	180	Median: 74 (65–94)	39.4	1 January 2017 to 31 December 2018	Last week	Cancer (100): Digestive organs (31.7) Respiratory and intrathoracic organs (18.8) Male genital organs (10) Lymphoid and haematopoietic (8.9) Female genital organs (7.8) Urinary tract (7.2) Melanoma and other skin (4.4) Breast (4.4) Unspecified sites (3.3) Other (3.3)	HTN (11.4%) T2DM (6.0%) IHD (4.6%) AFib (4.3%) Dyslipidaemia (4.1%)	STOPPFrail (V1 and V2), OncPal, PIP-CPC
Paque, 2018	Retrospective cohort, Register based cohort	Belgium (Europe)	74,368	Median: 86 (81–90)	56.7	28 February to 30 June 2012	Last 6 months, Last 4 months	Cancer (23.3) Non-cancer (76.7)	N/A	STOPPFrail V1
Pasina, 2018	Retrospective cohort, Hospice	Italy (Europe)	488	Mean: 79.7 (7.0)	47.5	March 2015 to February 2017	Last 6 months	Cancer (93.6) Non-cancer (6.4)	Median no. of comorbidities (IQR): 1 (0–2)	INTERCheck
Rausch, 2020	Retrospective cohort, Nursing home	Germany (Europe)	67,328	Mean: Dementia: 86.4 (6.5) No dementia: 84.6 (7.9)	69.8	1 January 2010 to 31 December 2014	Last 6 months	Dementia (43.15) No dementia (56.85)	N/A	Holmes criteria
Roux, 2019	Retrospective cohort, Hospital	France (Europe)	149	Mean: 82.1 (8.6)	46.3	1 January to 30 June 2014	Last 3 months	Cancer (62.4) Sepsis (20.1) Renal failure (26.3) Heart failure (25.0) Stroke (22.3) Haemorrhagic event (15.4) Thrombotic event (14.7) Alzheimer’s disease (14.8) MI (14.2) Other dementia (10.0) COPD (8.8) Respiratory failure (3.4) Parkinson’s disease (4.0) Peripheral vascular disease (2.7) Occlusive syndrome (2.0) Autolysis (0.7)	HTN (66.9%) T2DM (18.9%) Arthropathy (10.8%) Other chronic diseases (16.7%)	Todd criteria
Sakran, 2024	Retrospective cohort, Hospital	Israel (Asia)	155	Median: 69 (62–76)	45.7	August 2019–2021	Last 6 months	Cancer (100)	Not reported	2019 Beers Criteria, STOPP Criteria (2015), OncPal, DDI identified using Lexi-Interact Online, Micromedex, Deprescribing indications retrospectively classified using Helper-Strand Criteria for Drug Related Problems
Sourdet, 2018	Prospective cohort, Nursing home	France (Europe)	498	Mean: 88.8 (6.9)	69.5	May to June 2011	Last 6 months	MI (10.2) Peripheral vascular disease (18.9) Heart failure (28.1) HTN (52.8) Hemiplegia (4.4) Stroke (13.6) Dementia (48.6%) Chronic lung disease (15.5) Hepatic disease (2.0) Kidney disease (17.7) Cancer (19.9)	Depression (34.1%) T2DM (16.3%) Gastro-duodenal ulcer (4.2%)	Laroche criteria
Springer, 2020	Retrospective cohort, VA Nursing homes	United States (North America)	13,844	65–74 years (27.7%) 75–84 years (38.0%) ≥ 85 years (34.3%)	1.1	2009 to 2015	Last 6 months	Advanced dementia (30.4) Cancer (33.5) CAD or Stroke (not both) (75.5) CAD and Stroke (24.5)	T2DM (53.9%) CHF (52.9%) HTN (93.8%) Dyslipidaemia (70.4%) VTE (11.9%) AFib (19.1%)	N/A
Toscani, 2019	Retrospective cohort, Nursing home	Italy (Europe)	482	Median: 84.1 (79–88.7)	75.5	1 April 2013 to 31 January 2015	Last week	Advanced dementia (100): Alzheimer’s (26.8) Not defined (36.3) Vascular (28.6) Mixed (6.8) Lewy body (1.5)	Genitourinary (90.2%) Musculoskeletal (84.9%) Gastrointestinal tract (28.0%) Peripheral and central nervous system (65.1%) HTN (49.4%) Cardiovascular (49.2%) Head and neck (42.9%) Vascular (40.5%) Respiratory (20.7%) Endocrine-metabolic (28.0%) Kidney (11.4%) Liver (6.2%) Others (3.7%)	N/A

Studies marked ‘N/A’ did not use a formal PIM tool but operationalised specific medication classes as PIMs (e.g. statins [28], aspirin [29]) based on established criteria identifying medications of questionable benefit in end-of-life populations.

^a^Abbreviations: PIM, Potentially Inappropriate Medications; COPD, Chronic Obstructive Lung Disease; ESRD, End-Stage Renal Disease; CAD, Coronary Artery Disease; T2DM, Type 2 Diabetes Mellitus; HTN, Hypertension; IHD, Ischaemic Heart Disease; AFib, Atrial Fibrillation; CHF, Congestive Heart Failure; VTE, Venous Thromboembolism; MI, Myocardial Infarction; PPI, Proton-Pump Inhibitors.

### Summary of findings

#### Global prevalence of polypharmacy within the last 6 months of life

Five studies reported polypharmacy prevalence [28, 33, 36, 38, 43]. Overall, 76% (95% CI 65%–87%) of older adults received ≥5 medications in their final six months (Figure 2). Stratification showed that 41% (95% CI 31%–51%) received 5–9 medications and 34% (95% CI 9%–58%) received ≥10 medications (see Supplementary Appendix 6). Subgroup analyses showed no statistically significant differences by region or setting (see Supplementary Appendix 6). Doi plot showed asymmetry (LFK index = 6.63), indicating small-study effects (see Supplementary Appendix 7).

**Figure 2:**
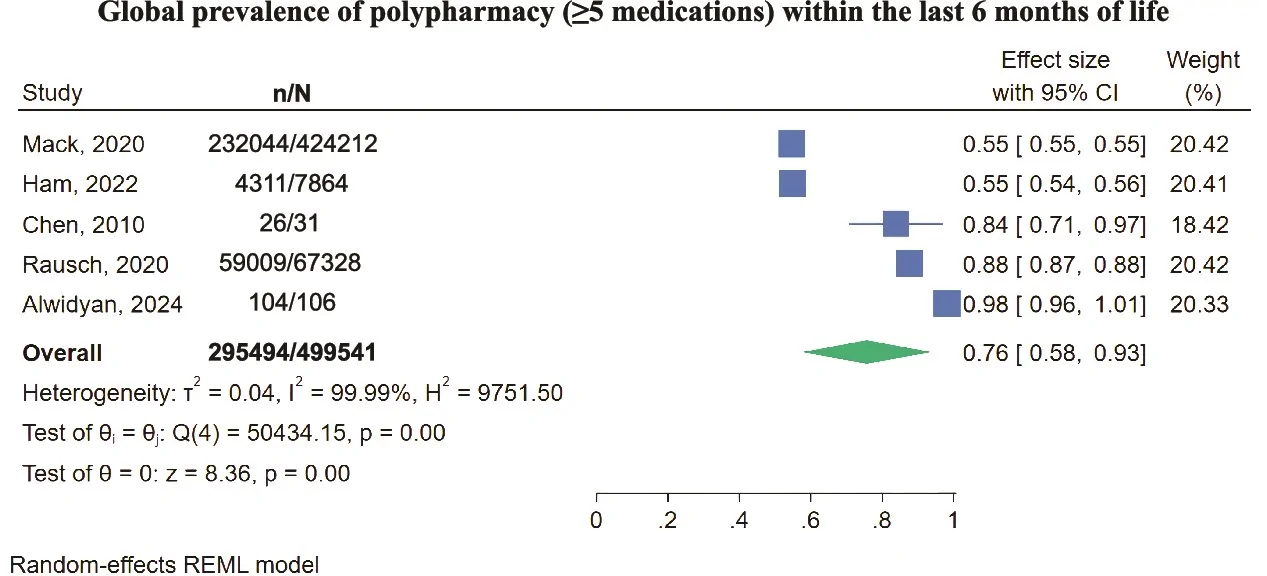
Global prevalence of polypharmacy (≥5 medications) within the last 6 months of life.

#### Medication burden at end-of-life

Eleven studies reported medication burden outcomes, including mean [32, 33, 35, 36, 39, 43–45] or median medication counts [12, 31, 40] (see Supplementary Appendix 6). The reported trajectory of medication burden near death was not uniform across studies. While Murphy et al. reported a notable increase from a median of 5–11 medications on the final day, other studies showed similar counts or a decline in the final days of life [31].

#### Global prevalence of PIM at the end of life

Fifteen studies reported PIM prevalence, including studies that did not use a formal PIM tool but operationalised specific medication classes as PIMs based on established criteria identifying medications of questionable benefit in end-of-life populations [28, 29]. Hattori et al. was not included in the pooled meta-analysis as prevalence data for individual PIM classes could not be extracted for the meta-analysis (see Supplementary Appendices 9 and 10) [41]. Overall, 54% (95% CI 40%–68%) of older adults used PIMs in their final six months (Figure 3). Prevalence remained similar across time points: last 6 months (56%), last 4 months (56%), last month (59%), with slight decrease in last week (49%) and day of death (39%) without statistically significant difference (Qb(4) = 7.44, *P* = .11; see Supplementary Appendix 8).

**Figure 3:**
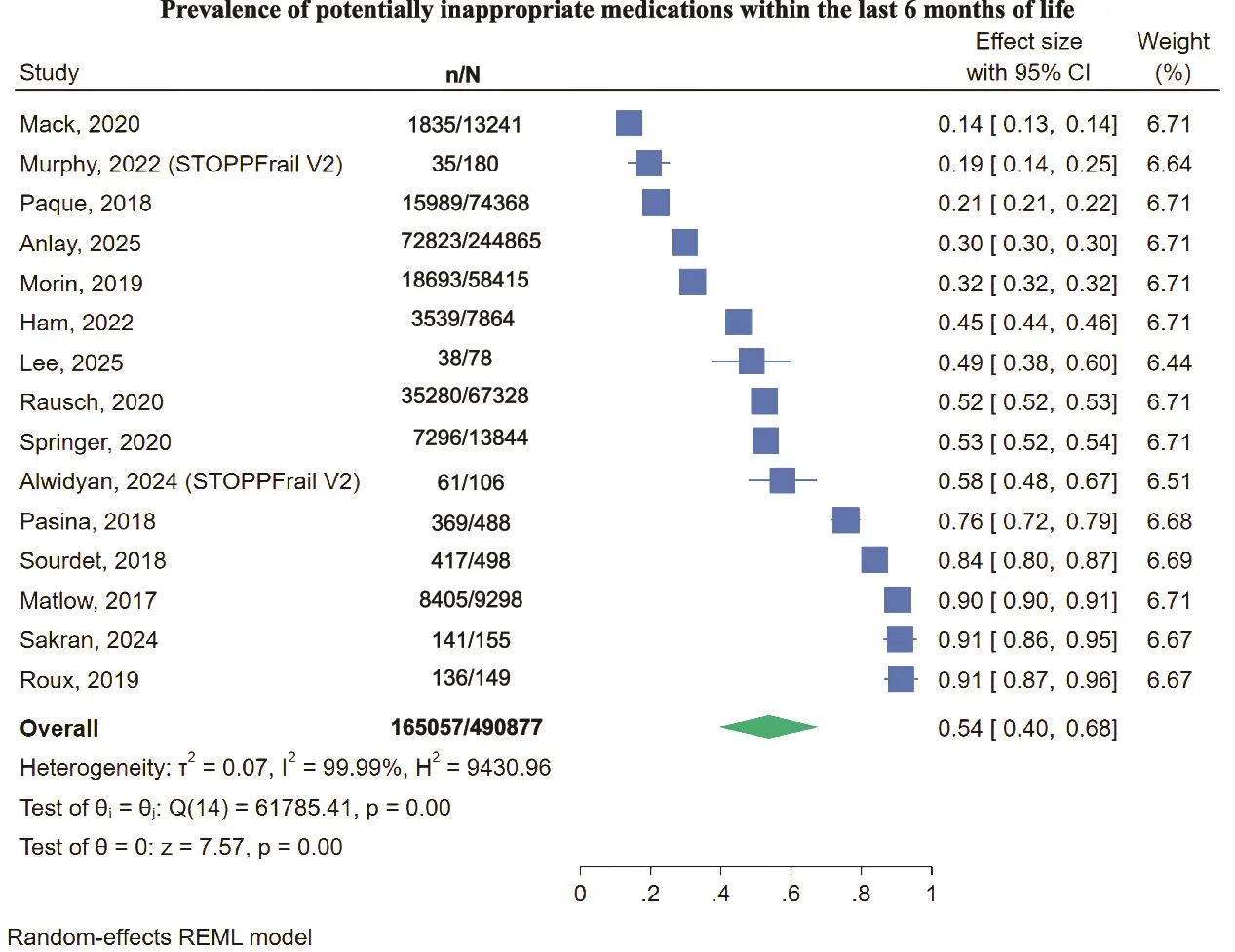
Prevalence of potentially inappropriate medications (PIM) within the last 6 months of life.

When stratified by setting, PIM prevalence was highest in hospitals (67%; 95% CI 20%–114%), followed by hospices (61%; 45%–77%), nursing homes (59%; 32%–85%) and registries (32%; 23%–42%) with a statistically significant difference (Qb(3) = 12.47, *P* = .01). By region, Asia had the highest prevalence (70%; 29%–112%), followed by North America (52%; 9%–96%) and Europe (51%; 35%–67%), but this was not statistically significant (Qb(2) = 0.73, *P* = .69).

When stratified by PIM tool type, studies using general geriatric criteria (Laroche criteria [39]; INTERCheck [35]) reported a higher pooled PIM prevalence of 80% (95% CI 72%–88%), compared to studies using palliative/end-of-life-specific criteria (STOPPFrail V1/V2, OncPal, PIP-CPC, Morin criteria, Holmes criteria, Todd criteria), which reported a 45% (95% CI 31%–60%). Test of group differences was statistically significant (Qb(1) = 16.81, *P* < .001), highlighting the substantial impact of tool selection on PIM prevalence estimates. Sakran *et al*. [12] was excluded from this analysis as they used a combination of Beers, STOPP and OncPal criteria and did not report separate prevalence estimates by individual tool.

When stratified by study population, studies that included predominantly cancer patients (≥90% cancer, *n* = 8) reported a pooled PIM prevalence of 52% (95% CI 36%–69%) compared to 55% (30%–80%) in non-cancer/mixed studies (*n* = 7), with no differences between both groups. Sensitivity analyses excluding studies with <5000 participants or moderate risk of bias yielded lower PIM prevalence; while excluding studies with <10% female did not change estimates (see Supplementary Appendix 8). Doi plot and LFK index (6.44) indicated major asymmetry, suggesting publication bias (see Supplementary Appendix 7).

#### Common PIM medication classes

Proton pump inhibitors (PPIs) were the most commonly flagged preventive PIM class, with descriptive pooled averages of almost 40% at six and four months before death, declining to 7.8% in the final week but rising to 42% on the day of death. Lipid-modifying agents showed a pooled average of approximately 33%, with marginal decline followed by a rebound in the final week. Antithrombotics use meanwhile persisted until the day of death. Antidementia medications showed a descriptive peak at 41.4% in the last 4 months, declining to 31.7% in the last week. Cardiovascular agents such as ACEi/ARB and supplements such as vitamins declined over time to nearly zero on the day of death (see Supplementary Appendix 9).

For symptomatic medication classes, averages showed different patterns. At 6 months before death, H2 antagonists showed the highest descriptive prevalence in one study (68.8%) [41], followed by PPI (24.5%), beta-blockers (19.1%), diuretics (17.2%), NSAIDs (16.8%), psycholeptics and oral steroids (both 14.4%). At 4 months, psycholeptics (31.1%), oral steroids (31.1%) and GI antispasmodics (26.6%) showed higher descriptive prevalence. By the day of death, most classes declined, although beta-blockers (5.3%), insulin (3.3%) and diuretics (2.2%) use persisted (see Supplementary Appendix 10).

#### Prevalence of potential prescribing omissions within the last 6 months of life

Only two studies assessed potential prescribing omissions (PPOs) (see Supplementary Appendix 11), precluding pooled analyses [31, 34]. In the study by Murphy and colleagues, they reported that 30% (54/180) of patients receiving opioids were not co-prescribed prophylactic laxatives as recommended by the PIP-CPC criteria. Conversely, Lee and colleagues reported that 76.6% of patients with moderate-to-severe cancer pain did not receive a sufficiently strong opioids as recommended by the WHO guidelines.

## Discussion

In this systematic review and meta-analysis, polypharmacy and PIM use were highly prevalent among older adults with a prognosis of ≤6 months. Three-quarters had polypharmacy, one-third had excessive polypharmacy (≥10 medications) and half used PIMs. PIM prevalence remained >50% until the final week of life, driven largely by continuation of preventive medications whose time-to-benefit exceeds life expectancy. These findings align with general geriatric populations, where polypharmacy prevalence ranges from 30% to 90% [46]. Similar nursing home studies used a cutoff of ≥9 medications, whereas studies included in this review used a stricter cutoff of ≥10 medications for excessive polypharmacy. Even so, nearly one in three older adults met this criterion. These rates mirror those reported in other long-term care settings, which range between 18.5% and 82.6% using the Beers criteria [47, 48].

However, our study noted that PIM prevalence varies substantially depending on assessment tool used. For instance, using the STOPPFrail V1 criteria gave a 23% PIM prevalence while the Laroche criteria was reported to be as high as 91%. This is probably because general geriatric tools such as Laroche and INTERCheck will flag some anticholinergics, cardiovascular preventives and psycholeptics as potentially inappropriate for robust older adults due to fall or cognitive risks [32, 35, 39]. However, in end-of-life care, these medications may be appropriate for symptom palliation [49]. As such, tools designed for limited prognosis (STOPPFrail, Morin criteria, OncPal) were more specific to identify medications that are generally non-beneficial [25, 31, 44]. This may explain the 35 percentage-point difference in PIM prevalence between general geriatric tools (80%) and palliative-specific tools (45%) observed in this study.

Across studies, medications appropriateness was judged variably, depending on the temporal relationship between benefit and prognosis. For instance, the use of PPIs and H2 receptor antagonists were classified as PIMs when used prophylactically without a clear indication [31, 32, 35, 45], while one study [41] grouped them with symptomatic medications. This wide variation across definition and tools highlights the ‘measurement paradox’, where prevalence estimates depend substantially on the tools selected, complicating cross-study comparisons and quality measurement in palliative care. This suggest that the results should be interpreted with caution, but also highlights the need for a standardised, consensus-based instrument for assessing PIM in this population [50]. Another important observation from our study was that only few studies have attempted to examine the rates of PPO. This may be because PPO identification requires prospective study designs with direct patient assessment, whereas PIMs can often be assessed retrospectively.

Temporal analysis also revealed an opportunity for deprescribing, as PIM prevalence remained high, at ~56% until 1 month before death, and only dropping to 29% in the final week before death. Importantly, preventive therapies were often used until an acute deterioration or dysphagia rather than being proactively deprescribed when treatment goals shifted to comfort-focused care. Studies to date have shown that reducing medication burden through deprescribing can substantially improve quality of life for older adults nearing the end of life, by minimising side effects, reducing pill burden and aligning treatment with comfort-focused goals [27, 49]. As decisions about medication continuation near the end-of-life are inherently complex, it must be grounded in patient-centred care and shared decision-making [51]. For example, some patients with a life expectancy of several months may choose to continue anticoagulants or antiplatelet agents to reduce the risk of a disabling stroke that could severely impair quality of life during their remaining time, particularly in the context of cancer-associated prothrombotic states [2, 3]. Similarly, patients with diabetes may prefer to continue oral hypoglycaemics to avoid symptomatic hyperglycaemia, even if the long-term microvascular benefits are unlikely to materialise [5]. These nuanced considerations underscore that deprescribing is not a one-size-fits-all approach, but rather a shared decision-making process that requires ongoing dialogue between clinicians, patients and families [50, 51].

### Strengths and limitations

This study offers several strengths. To our best knowledge, this is the first study to comprehensively summarise polypharmacy, PIM and PPO prevalence in older adults with a uniformly defined prognosis of ≤6 months, establishing a baseline for future interventions. The review followed PRISMA guidelines. However, there are several limitations that needs to be taken into consideration. Firstly, there is substantial small-study effects as most doi plots shows asymmetry. Sensitivity analyses which were restricted to large registry-based studies gave lower estimates for both polypharmacy and PIMs, suggesting that the true population prevalence may be more conservative than those reported in this study. There were also limited data from low- and middle-income countries. Additionally, the use of AND operator between closely related search concepts (‘life-limiting illness’ and ‘end-of-life care’) may have limited search sensitivity. Although we mitigated this by hand-searching reference lists of included studies, some relevant studies may have been inadvertently missed, potentially introducing selection bias. PPO data were too limited for pooled analysis.

Excessive polypharmacy could only be examined at thresholds reported (≥10 or ≥ 15), precluding direct analysis at the recommended ≥9 cut-off for nursing homes [13]. Furthermore, we did not search grey literature, which may have introduced publication bias by excluding unpublished studies or conference abstracts. However, the inclusion of large registry-based, peer-reviewed studies (*n* ≥ 5000) may partially offset this limitation. Descriptive comparisons of PIM prevalence across time points should also be interpreted with caution, as these estimates are derived from different studies rather than from longitudinal follow-up of the same cohorts.

### Implications

Inappropriate prescribing is common in long-term care [48]. Despite guidelines recommending bi-annual medication reviews [52], PIMs persist until the final weeks of life, suggesting the need for more regular reviews in this population. These medication reviews should ideally be performed during transitions of care such as during hospice enrolment or when patient’s goal changes, with proactive deprescribing. Finally, a PIM identification tool for those at the end-of-life is needed given the lack of specificity of most currently available tools.

## Conclusion

Polypharmacy (≥5 medications) was present in three-quarters of older adults with a prognosis of 6 months and less, and half received PIMs. Preventive medications were frequently continued until the final days. This highlights an urgent need for guidelines to facilitate proactive, goal-concordant deprescribing interventions in palliative care.

## Supplementary Material

aa-26-1774-File005_afag326

## Data Availability

All data are from published studies cited in this article. Extracted data are available from the corresponding author on reasonable request. PROSPERO: CRD42023454636.

## References

[1] McNeil MJ, Kamal AH, Kutner JS et al. The burden of polypharmacy in patients near the end of life. J Pain Symptom Manage 2016;51:178–183.e2. 10.1016/j.jpainsymman.2015.09.003. PMC4733587 26432571

[2] Narayan SW, Nishtala PS. Discontinuation of preventive medicines in older people with limited life expectancy: a systematic review. Drugs Aging 2017;34:767–76. 10.1007/s40266-017-0487-1. 28853000

[3] Poudel A, Yates P, Rowett D et al. Use of preventive medication in patients with limited life expectancy: a systematic review. J Pain Symptom Manage 2017;53:1097–1110.e1. 10.1016/j.jpainsymman.2016.12.350. 28192226

[4] Organization, T.N.H.A.P.C . Home, 2024. https://www.nhpco.org/

[5] Hughes C . Appropriate and inappropriate polypharmacy—choosing the right strategy. Br J Clin Pharmacol 2021;87:84–6. 10.1111/bcp.14589. 33025612

[6] Jamil YA, Cohen R, Alameddine DK et al. Cholesterol lowering in older adults: should we wait for further evidence? Curr Atheroscler Rep 2024;26:521–36. 10.1007/s11883-024-01224-4. 38958924

[7] Puig T, Leache L, González-Senac NM et al. Prevalence of potentially inappropriate medications and prescription dynamics in elderly hospitalized patients in Spain. BMC Geriatr 2024;24:798. 10.1186/s12877-024-05308-3. PMC11443693 39350081

[8] Organization, W.H . Palliative Care, 2020. https://www.who.int/news-room/fact-sheets/detail/palliative-care

[9] Shrestha R, Shaw E, Mullen L et al. Outcomes of deprescribing for people with life-limiting conditions: a systematic review. Palliat Med 2026;40:490–513. 10.1177/02692163261416281. PMC13062472 41724578

[10] Moher D, Liberati A, Tetzlaff J et al. Preferred reporting items for systematic reviews and meta-analyses: the PRISMA statement. PLoS Med 2009;6:e1000097. 10.1371/journal.pmed.1000097. PMC2707599 19621072

[11] Page MJ, McKenzie JE, Bossuyt PM et al. The PRISMA 2020 statement: an updated guideline for reporting systematic reviews. Int J Surg 2021;88:105906. 10.1016/j.ijsu.2021.105906. 33789826

[12] Sakran R, Litvak M, Haim N et al. Deprescribing in hospitalized patients with cancer: a clinical pharmacist-initiated multidisciplinary intervention. J Oncol Pharm Pract 2024;32:490–7. 10.1177/10781552241294016. 39469991

[13] Jokanovic N, Tan ECK, Dooley MJ et al. Prevalence and factors associated with polypharmacy in long-term care facilities: a systematic review. J Am Med Dir Assoc 2015;16:535.e1–12. 10.1016/j.jamda.2015.03.003. 25869992

[14] Moola S, Munn Z, Tufanaru C et al. Chapter 7: systematic reviews of etiology and risk. JBI Manual for Evidence Synthesis 2020; https://synthesismanual.jbi.global/.

[15] Wells GA, Shea B, O'Connel D. The Newcastle-Ottawa Scale (NOS) for Assessing the Quality of Nonrandomised Studies in Meta-Analyses, 2009. http://www.ohri.ca/programs/clinical_epidemiology/oxford.htm

[16] DerSimonian R, Laird N. Meta-analysis in clinical trials. Control Clin Trials 1986;7:177–88. 10.1016/0197-2456(86)90046-2. 3802833

[17] By the American Geriatrics Society Beers Criteria® Update Expert, P . American Geriatrics Society 2019 updated AGS beers criteria® for potentially inappropriate medication use in older adults. J Am Geriatr Soc 2019;67:674–94. 10.1111/jgs.15767. 30693946

[18] Laroche ML, Charmes JP, Merle L. Potentially inappropriate medications in the elderly: a French consensus panel list. Eur J Clin Pharmacol 2007;63:725–31. 10.1007/s00228-007-0324-2. 17554532

[19] Ghibelli S, Marengoni A, Djade CD et al. Prevention of inappropriate prescribing in hospitalized older patients using a computerized prescription support system (INTERcheck(®)). Drugs Aging 2013;30:821–8. 10.1007/s40266-013-0109-5. 23943248

[20] O'Mahony D, O'Sullivan D, Byrne S et al. STOPP/START criteria for potentially inappropriate prescribing in older people: version 2. Age Ageing 2015;44:213–8. 10.1093/ageing/afu145. PMC4339726 25324330

[21] Holmes HM, Sachs GA, Shega JW et al. Integrating palliative medicine into the Care of Persons with advanced dementia: identifying appropriate medication use. J Am Geriatr Soc 2008;56:1306–11. 10.1111/j.1532-5415.2008.01741.x. 18482301

[22] Morin L, Laroche ML, Vetrano DL et al. Adequate, questionable, and inadequate drug prescribing for older adults at the end of life: a European expert consensus. Eur J Clin Pharmacol 2018;74:1333–42. 10.1007/s00228-018-2507-4. PMC6132505 29934849

[23] Lindsay J, Dooley M, Martin J et al. The development and evaluation of an oncological palliative care deprescribing guideline: the 'OncPal deprescribing guideline. Support Care Cancer 2015;23:71–8. 10.1007/s00520-014-2322-0. 24975044

[24] Cadogan CA, Murphy M, McLean S et al. Development of criteria for identifying potentially inappropriate prescribing in older adults with cancer receiving palliative care (PIP-CPC). J Geriatr Oncol 2021;12:1193–9. 10.1016/j.jgo.2021.06.003. 34144924

[25] Lavan AH, Gallagher P, Parsons C et al. STOPPFrail (screening tool of older persons prescriptions in frail adults with limited life expectancy): consensus validation. Age Ageing 2017;46:600–7. 10.1093/ageing/afx005. 28119312

[26] Curtin D, Gallagher P, O'Mahony D. Deprescribing in older people approaching end-of-life: development and validation of STOPPFrail version 2. Age Ageing 2021;50:465–71. 10.1093/ageing/afaa159. 32997135

[27] Todd A, Husband A, Andrew I et al. Inappropriate prescribing of preventative medication in patients with life-limiting illness: a systematic review. BMJ Support Palliat Care 2017;7:113–21. 10.1136/bmjspcare-2015-000941. 26733578

[28] Mack DS, Tjia J, Hume AL et al. Prevalent statin use in long-stay nursing home residents with life-limiting illness. J Am Geriatr Soc 2020;68:708–16. 10.1111/jgs.16336. PMC8072406 32057091

[29] Springer SP, Mor MK, Sileanu F et al. Incidence and predictors of aspirin discontinuation in older adult veteran nursing home residents at end of life. J Am Geriatr Soc 2020;68:725–35. 10.1111/jgs.16346. PMC7837284 32052858

[30] Furuya-Kanamori L, Barendregt JJ, Doi SAR. A new improved graphical and quantitative method for detecting bias in meta-analysis. Int J Evid Based Healthc 2018;16:195–203. 10.1097/XEB.0000000000000141. 29621038

[31] Murphy M, Bennett K, Wright M et al. Potentially inappropriate prescribing in older adults with cancer receiving specialist palliative care: a retrospective observational study. Int J Clin Pharm 2023;45:174–83. 10.1007/s11096-022-01506-4. PMC9664032 36378404

[32] Roux B, Morin L, Papon A et al. Prescription and deprescription of medications for older adults receiving palliative care during the last 3 months of life: a single-center retrospective cohort study. Eur Geriat Med 2019;10:463–71. 10.1007/s41999-019-00175-3. 34652792

[33] Alwidyan T, McCorry NK, Black C et al. Prescribing and deprescribing in older people with life-limiting illnesses receiving hospice care at the end of life: a longitudinal, retrospective cohort study. Palliat Med 2024;38:121–30. 10.1177/02692163231209024. PMC10798021 38032069

[34] Lee J, Lee CW, Kim HS et al. Potentially inappropriate prescriptions in end-of-life cancer patients in home-based hospice care. J Pain Symptom Manage 2025;70:22–9. 10.1016/j.jpainsymman.2025.03.021. 40147504

[35] Pasina L, Recchia A, Agosti P et al. Prevalence of preventive and symptomatic drug treatments in hospice care: an Italian observational study. Am J Hosp Palliat Med 2019;36:216–21. 10.1177/1049909118794926. 30114944

[36] Chen IC, Liu ML, Twu FC et al. Use of medication by nursing home residents nearing end of life: a preliminary report. J Nurs Res 2010;18:199–205. 10.1097/JNR.0b013e3181ed5731. 20808079

[37] Matlow JN, Bronskill SE, Gruneir A et al. Use of medications of questionable benefit at the end of life in nursing home residents with advanced dementia. J Am Geriatr Soc 2017;65:1535–42. 10.1111/jgs.14844. 28369696

[38] Rausch C, Hoffmann F. Prescribing medications of questionable benefit prior to death: a retrospective study on older nursing home residents with and without dementia in Germany. Eur J Clin Pharmacol 2020;76:877–85. 10.1007/s00228-020-02859-3. PMC7239800 32219538

[39] Sourdet S, Rochette C, de Souto Barreto P et al. Drug prescriptions in nursing home residents during their last 6 months of life: data from the IQUARE study. J Nutr Health Aging 2018;22:904–10. 10.1007/s12603-018-1071-z. PMC12876287 30272091

[40] Toscani F, Finetti S, Giunco F et al. The last week of life of nursing home residents with advanced dementia: a retrospective study. BMC Palliat Care 2019;18:117. 10.1186/s12904-019-0510-x. PMC6935223 31882007

[41] Hattori Y, Hamada S, Iwagami M et al. Current status of drug prescribing among older adults with advanced-stage cancer receiving home medical Care in Japan: a Nationwide study. JMA J 2025;8:1350–8. 10.31662/jmaj.2025-0179. PMC12598258 41220503

[42] Anlay DZ, Paque K, van den Eynden B et al. International deprescribing guidelines did not impact actual practice in deprescribing of potentially inappropriate medications for nursing home residents: an interrupted time series analysis. Drugs Aging 2025;42:485–99. 10.1007/s40266-025-01197-2. 40113742

[43] Ham L, Geijteman ECT, Aarts MJ et al. Use of potentially inappropriate medication in older patients with lung cancer at the end of life. J Geriat Oncol 2022;13:53–9. 10.1016/j.jgo.2021.07.009. 34366274

[44] Morin L, Wastesson JW, Laroche ML et al. How many older adults receive drugs of questionable clinical benefit near the end of life? A cohort study. Palliat Med 2019;33:1080–90. 10.1177/0269216319854013. PMC6691599 31172885

[45] Paque K, de Schreye R, Elseviers M et al. Discontinuation of medications at the end of life: a population study in Belgium, based on linked administrative databases. Br J Clin Pharmacol 2019;85:827–37. 10.1111/bcp.13874. PMC6422643 30667540

[46] Andrade A, Nascimento T, Cabrita C et al. Potentially inappropriate medication: a pilot study in institutionalized older adults. Healthcare 2024;12:1275. 10.3390/healthcare12131275. PMC11241476 38998810

[47] Gaubert-Dahan M-L, Sebouai A, Tourid W et al. The impact of medication review with version 2 STOPP (screening tool of older Person's prescriptions) and START (screening tool to alert doctors to right treatment) criteria in a French nursing home: a 3-month follow-up study. Ther Adv Drug Saf 2019;10:2042098619855535. 10.1177/2042098619855535. PMC6558549 31217947

[48] Storms H, Marquet K, Aertgeerts B et al. Prevalence of inappropriate medication use in residential long-term care facilities for the elderly: a systematic review. Eur J Gen Pract 2017;23:69–77. 10.1080/13814788.2017.1288211. PMC5774291 28271916

[49] Cadogan CA, Murphy M, Boland M et al. Prescribing practices, patterns, and potential harms in patients receiving palliative care: a systematic scoping review. Explor Res Clin Soc Pharm 2021;3:100050. 10.1016/j.rcsop.2021.100050. PMC9031741 35480601

[50] Dreischulte T, Sanftenberg L, Hennigs P et al. Detecting medication risks among people in need of care: performance of six instruments. Int J Environ Res Public Health 2023;20:2327. 10.3390/ijerph20032327. PMC9915255 36767705

[51] Yeh A, Sun AZ, Chernicoff H. Polypharmacy in hospice and palliative care. Clin Geriatr Med 2022;38:693–704. 10.1016/j.cger.2022.05.009. 36210085

[52] Mays AM, Saliba D, Feldman S et al. Quality indicators of primary care provider engagement in nursing home care. J Am Med Dir Assoc 2018;19:824–32. 10.1016/j.jamda.2018.08.001. 30268288

