# Supplementary material for: Prevalence of polypharmacy and potentially inappropriate prescribing in older adults with a prognosis of 6 months or less: a systematic review and meta-analysis
Source: Age Ageing. 2026 Nov 13;55(11):afag326. doi: 10.1093/ageing/afag326 (PMC13765224; doi:10.1093/ageing/afag326)
Supplement: Supplementary file 1 [file aa-26-1774-file005_afag326.docx]

**Prevalence of Polypharmacy and Potentially Inappropriate Prescribing in Older Adults with a Prognosis of Six Months or Less: A Systematic Review and Meta-analysis**

**(APPENDIX)**

**Table of Contents (Appendix)**

**Appendix 1: PRISMA Checklist** (Page 1-6)

**Appendix 2: Search strategy** (Page 7-10)

**Appendix 3: List of excluded studies** (Page 11-15)

**Appendix 4: Study Findings, Limitations and Recommendations across Included Articles** (Page 16-22)

**Appendix 5: Methodological quality appraisal of included studies** (Page 23-25)

**Appendix 6: Subgroup and Sensitivity analyses of Polypharmacy Prevalence** (Page 26-31)

Note: The ≥10 threshold approximates but likely underestimates the prevalence of excessive polypharmacy at the ≥9 cut-off recommended for nursing homes (Jokanovic et al., 2015).

**Appendix 7: Publication bias assessments** (Page 32-34)

**Appendix 8: Subgroupand Sensitivityanalyses of Potentially Inappropriate Medication (PIM) Prevalence** (Page 35-45)

**Appendix 9: Top 10 Drug Classes for Preventive Medications Identified as PIM at each time points** (Page 46-48)

**Appendix 10: Top 10 Drug Classes for Symptomatic Medications Identified as PIM at each time points** (Page 49-50)

**Appendix 11: Prevalence of Potential Prescribing Omissions (PPO)** (Page 51)

**PRISMA 2020 Checklist**

| **Section and Topic** | **Item #** | **Checklist item** | **Location where item is reported** |
| --- | --- | --- | --- |
| **TITLE** | | |  |
| Title | 1 | Identify the report as a systematic review. | Title page: “A Systematic Review and Meta-analysis” lines 1-2 |
| **ABSTRACT** | | |  |
| Abstract | 2 | See the PRISMA 2020 for Abstracts checklist. | Abstract section, page 1, lines 11-  38; page 2, lines  1-7 |
| **INTRODUCTION** | | |  |
| Rationale | 3 | Describe the rationale for the review in the context of existing knowledge. | Introduction, page 2, lines 38-62 |
| Objectives | 4 | Provide an explicit statement of the objective(s) or question(s) the review addresses. | Introduction, final paragraph, page 2, lines 63-65 |
| **METHODS** | | |  |
| Eligibility criteria | 5 | Specify the inclusion and exclusion criteria for the review and how studies were grouped for the syntheses. | Methods: “Eligibility criteria”, page 3, lines 95-  115 |
| Information sources | 6 | Specify all databases, registers, websites, organisations, reference lists and other sources searched or consulted to identify studies. Specify the date when each source was last searched or consulted. | Methods: “Search strategy” page 3,  lines 78-82 |
| Search strategy | 7 | Present the full search strategies for all databases, registers and websites, including any filters and limits used. | Appendix 2 (full search string and MeSH terms provided) |
| Selection process | 8 | Specify the methods used to decide whether a study met the inclusion criteria of the review, including how many reviewers screened each record and each report retrieved, whether they worked independently, and if applicable, details of automation tools used in the process. | Methods: “Study selection, data extraction and quality appraisal”, page 4, lines 135-  142 |
| Data collection process | 9 | Specify the methods used to collect data from reports, including how many reviewers collected data from each report, whether they worked independently, any processes for obtaining or confirming data from study investigators, and if applicable, details of automation tools used in the process. | Methods: “Study selection, data extraction and quality appraisal”, page 4, lines 135-  142 |
| Data items | 10a | List and define all outcomes for which data were sought. Specify whether all results that were compatible with each outcome domain in each study were sought (e.g. for all measures, time points, analyses), and if not, the methods used to decide which results to collect. | Methods: “Data synthesis”, page 4, lines 155-165; results reported in Figure 2 (polypharmacy)  and Figure 3 **1** |

**PRISMA 2020 Checklist**

| **Section and Topic** | **Item #** | **Checklist item** | **Location where item is reported** |
| --- | --- | --- | --- |
|  |  |  | (PIMs) |
|  | 10b | List and define all other variables for which data were sought (e.g. participant and intervention characteristics, funding sources). Describe any assumptions made about any missing or unclear information. | Table 1, page 5-8; Methods: authors contacted for missing data (page 4, line 139) |
| Study risk of bias assessment | 11 | Specify the methods used to assess risk of bias in the included studies, including details of the tool(s) used, how many reviewers assessed each study and whether they worked independently, and if applicable, details of automation tools used in the process. | Methods: “Study selection, data extraction and quality appraisal”, page 4, lines 141-  142; Appendix 5  (page 77-79) |
| Effect measures | 12 | Specify for each outcome the effect measure(s) (e.g. risk ratio, mean difference) used in the synthesis or presentation of results. | Methods: “Data synthesis” page 4,  lines 155-159 |
| Synthesis methods | 13a | Describe the processes used to decide which studies were eligible for each synthesis (e.g. tabulating the study intervention characteristics and comparing against the planned groups for each synthesis (item #5)). | Methods: “Data synthesis”, page 4, lines 155-159 |
|  | 13b | Describe any methods required to prepare the data for presentation or synthesis, such as handling of missing summary statistics, or data conversions. | Methods: “Study selection, data extraction and quality appraisal” -  - authors contacted for missing data, page 4, line 139 |
|  | 13c | Describe any methods used to tabulate or visually display results of individual studies and syntheses. | Results: Figures 1-3,  Appendix 4-11,  Tables 1-2 |
|  | 13d | Describe any methods used to synthesize results and provide a rationale for the choice(s). If meta-analysis was performed, describe the model(s), method(s) to identify the presence and extent of statistical heterogeneity, and software package(s) used. | Methods: “Data synthesis”, page 4, lines 155-160; heterogeneity reported with I^2^ in Appendix 6 and 8 |
|  | 13e | Describe any methods used to explore possible causes of heterogeneity among study results (e.g. subgroup analysis, meta-regression). | Methods: “Data synthesis”, page 4, lines 160-161; stratification by setting, region, and assessment tool presented in Appendix 6 and 8 |
|  | 13f | Describe any sensitivity analyses conducted to assess robustness of the synthesized results. | Methods: “Data synthesis”, page 4, lines 160-161;  Results: page 12,  lines 242-247; **2** |

**PRISMA 2020 Checklist**

| **Section and Topic** | **Item #** | **Checklist item** | **Location where item is reported** |
| --- | --- | --- | --- |
|  |  |  | Appendix 8 (tool type sensitivity analysis) |
| Reporting bias assessment | 14 | Describe any methods used to assess risk of bias due to missing results in a synthesis (arising from reporting biases). | Methods: “Data synthesis”, page 4, lines 162;  Appendix 7 (Doi plots and LFK indices) |
| Certainty assessment | 15 | Describe any methods used to assess certainty (or confidence) in the body of evidence for an outcome. | Not formally assessed using GRADE  (prevalence meta-analysis).  Certainty considered qualitatively via risk of bias, heterogeneity, imprecision, and publication bias (Methods, page 4,  lines 159-160; Discussion, page 19, lines 563-587) |
| **RESULTS** | | |  |
| Study selection | 16a | Describe the results of the search and selection process, from the number of records identified in the search to the number of studies included in the review, ideally using a flow diagram. | Results: “Search results”, page 5,  lines 177-182;  Figure 1, page 20,  lines 612-613 |
|  | 16b | Cite studies that might appear to meet the inclusion criteria, but which were excluded, and explain why they were excluded. | Results: page 5,  lines 178-180;  Appendix 3 (completed list of excluded studies with reasons) |
| Study characteristics | 17 | Cite each included study and present its characteristics. | Table 1, page 5-8;  Appendix 4 |
| Risk of bias in studies | 18 | Present assessments of risk of bias for each included study. | Appendix 5, page  77-79; discussion of quality in Results, page 8,  lines 148-150 |
| Results of individual studies | 19 | For all outcomes, present, for each study: (a) summary statistics for each group (where appropriate) and (b) an effect estimate and its precision (e.g. confidence/credible interval), ideally using structured tables or plots. | Appendix 6, page  13; individual study results are  presented in **_3_** |

**PRISMA 2020 Checklist**

| **Section and Topic** | **Item #** | **Checklist item** | **Location where item is reported** |
| --- | --- | --- | --- |
|  |  |  | narrative synthesis and tables |
| Results of syntheses | 20a | For each synthesis, briefly summarise the characteristics and risk of bias among contributing studies. | Results: pages 8-  13; summary of contributing studies in Table 1, page 5-8, and narrative description, page 5, lines 183-186 |
|  | 20b | Present results of all statistical syntheses conducted. If meta-analysis was done, present for each the summary estimate and its precision (e.g. confidence/credible interval) and measures of statistical heterogeneity. If comparing groups, describe the direction of the effect. | Results: Figure 2,  page 8 (polypharmacy); Figure 3, page 11 (PIM);  heterogeneity reported in Appendix 6 and 8 |
|  | 20c | Present results of all investigations of possible causes of heterogeneity among study results. | Results: Table 2,  page 9 (polypharmacy by region/setting); Appendix 6 and 8 (subgroup and sensitivity analyses including tool type sensitivity analysis); page 12, lines 242-247 |
|  | 20d | Present results of all sensitivity analyses conducted to assess the robustness of the synthesized results. | Results: page 12,  lines 248-250;  Appendix 8 (sensitivity analyses excluding studies with <5,000 participants, moderate risk of bias, <10% female; tool type sensitivity analysis) |
| Reporting biases | 21 | Present assessments of risk of bias due to missing results (arising from reporting biases) for each synthesis assessed. | Appendix 7, page  45-46;  polypharmacy LFK=6.63, PIM LFK=6.44  indicating major  asymmetry; **_4_** |

**PRISMA 2020 Checklist**

| **Section and Topic** | **Item #** | **Checklist item** | **Location where item is reported** |
| --- | --- | --- | --- |
|  |  |  | mentioned in Results, page 8, line 190 and page 12, line 250 |
| Certainty of evidence | 22 | Present assessments of certainty (or confidence) in the body of evidence for each outcome assessed. | Not formally GRADE;  discussed qualitatively in Discussion (page 19, lines 563-587) and Methods (page 4, lines 159-  160) |
| **DISCUSSION** | | |  |
| Discussion | 23a | Provide a general interpretation of the results in the context of other evidence. | Discussion, page 14, lines 406-410 |
|  | 23b | Discuss any limitations of the evidence included in the review. | Discussion, “Strengths and limitations”, page 19, lines 563-587 |
|  | 23c | Discuss any limitations of the review processes used. | Discussion, “Strengths and limitations”, page  19, lines 563-587 |
|  | 23d | Discuss implications of the results for practice, policy, and future research. | Discussion, pages 18-19, lines 548-  560; Implications,  page 19, lines  590-594 |
| **OTHER INFORMATION** | | |  |
| Registration and protocol | 24a | Provide registration information for the review, including register name and registration number, or state that the review was not registered. | Methods, page 3,  lines 82-83; registration: PROSPERO CRD42023454636 |
|  | 24b | Indicate where the review protocol can be accessed, or state that a protocol was not prepared. | Protocol available from PROSPERO |
|  | 24c | Describe and explain any amendments to information provided at registration or in the protocol. | Methods, “Study registration”, page 3, lines 86-92 (deviations from registered protocol described) |
| Support | 25 | Describe sources of financial or non-financial support for the review, and the role of the funders or sponsors in the review. | Funding section, page 20, lines  596-598 |
| Competing interests | 26 | Declare any competing interests of review authors. | Conflicts of  Interest, page 20, **5** |

**PRISMA 2020 Checklist**

| **Section and Topic** | **Item #** | **Checklist item** | **Location where item is reported** |
| --- | --- | --- | --- |
|  |  |  | lines 606-608 |
| Availability of data, code and other materials | 27 | Report which of the following are publicly available and where they can be found: template data collection forms; data extracted from included studies; data used for all analyses; analytic code; any other materials used in the review. | Data available from corresponding author on reasonable request; no new data were generated (page 20, lines 596-608) |

*From:* Page MJ, McKenzie JE, Bossuyt PM, Boutron I, Hoffmann TC, Mulrow CD, et al. The PRISMA 2020 statement: an updated guideline for reporting systematic reviews. BMJ 2021;372:n71. doi: 10.1136/bmj.n71. This work is licensed under CC BY 4.0. To view a copy of this license, visit <https://creativecommons.org/licenses/by/4.0/>

**Appendix 2 - Search Strategy**

|  | **Population** | | | **Exposure** | | |
| --- | --- | --- | --- | --- | --- | --- |
| Main Concept | Older adults | Life-limiting illness | EOL care | Inappropriate Prescribing Issues | | |
|  |  |  |  | Polypharmacy | PIMs | PPOs |
| Synonyms, | Elder* | Terminal* ill* | Palliat* | Medication Burden | Inappropriate* Prescr* | Medic* Omission* |
| Spelling | Old* person* | End stage | Terminal Care | Conc* Medication | Inappropriate Medic* | Prescr* Omission* |
| variations | Old* people* | Late stage | Hospice* | Multimedication | Potentially Harmful Prescr* | Drug Omission* |
| (Google, | Old* adult* | Late phase | Comfort Care | Multimorbid* | Potentially Harmful Medic* | Omit* Medic* |
| Thesaurus, | Senior* | Terminal stage | Advanc* Care Planning | Comorbid* | Unsafe Medic* | Omit* Prescr* |
| dictionary | Veteran* | Incurable | Oncology | Polydrug* | Unsafe Prescr* | Omit* Drug* |
| etc.) | Golden age* | Advanc* stage | Approaching Death | Polymedication* | Deprescr* | Untreat* |
|  | Geriatr* | Irreversible | Near Death |  | Utili?ation | Undertreat* |
|  | Age* | Progressive decline | Actively Dying |  | Continuity of care | Miss* Medic* |
|  | Ag?ing | Chronic |  |  |  | Miss* Drug* |
|  | Retire* | Severe |  |  |  | Miss* Prescr* |
|  | Pensioner* | Limited life expectancy |  |  |  | Unfill* Medic* |
|  |  | Fatal |  |  |  | Unfill* Drug* |
|  |  | Limited prognosis |  |  |  | Unfill* Prescr* |
|  |  | End of life |  |  |  |  |
| Ovid | Aged | Death* | Tertiary Care Cent* | Polypharmacy | Potentially Inappropriate | Inappropriate |
| Medline | Age | Life expectancy | Cancer |  | Medic* | Prescr* |
| Medical | Ag?ing | Terminal* Ill* | Hospic* |  | Medica* Error* |  |
| Subject | Retire* | End stage | Terminal care |  | Malpractice* |  |
| Heading | Pension* | Incurable disease* | Palliat* |  | Practice pattern* |  |

| (MeSH) | Veteran* Geriatr* | Chronic disease* Fatal* | Nursing care Patient comfort  Advanc* care planning Oncology |  |  |  |
| --- | --- | --- | --- | --- | --- | --- |

**Search String:**

Concept 1: (Elder* OR Old OR Older OR Senior* OR Aged OR Aging OR Ageing) NOT (P*ediatric OR Child* OR Infant OR Adolescen*)

Concept 2: (Late stage OR Terminal stage OR Advanced stage OR Irreversible OR Progressive decline OR Limited prognosis OR Life expectancy OR End of life OR Terminal illness OR End stage OR Incurable disease OR Chronic disease)

Concept 3: (Comfort care OR Palliative care OR Terminal care OR Tertiary care OR End of life care OR Hospice care) OR (Cancer OR Oncology) OR (Pain management OR Symptom management OR Advance care planning)

Concept 4: (Medication Burden OR Drug Burden OR Polypharmacy OR Polydrug*) OR (Inappropriate* Prescr* OR Potentially Harmful Prescr* OR Potentially Harmful Medic* OR Unsafe Prescr* OR Deprescr* OR Potentially Inappropriate OR Medication Error OR Malpractice) OR (Omission* OR Omit OR Untreat* OR Miss* OR Unfill*)

Concept 5: Frequency OR Prevalence OR Rate OR Occurrence OR Incidence

Final Search String: Concept 1 AND Concept 2 AND Concept 3 AND Concept 4 AND Concept 5

**Database: PubMed**

Date searched: April 30, 2026 Limits: Humans, Aged 65+ years

Search Strategy:

#1 “polypharmacy”[MeSH Terms] OR “polypharmacy”[tiab] OR “medication burden”[tiab] OR “concurrent medication”[tiab] OR “multimedication”[tiab] OR “polymedication”[tiab] OR “multimorbidity”[tiab]

#2 “inappropriate prescribing”[MeSH Terms] OR “inappropriate prescribing”[tiab] OR “potentially inappropriate medication”[tiab] OR “potentially inappropriate medications”[tiab] OR “PIM”[tiab] OR “PIMs”[tiab] OR “inappropriate medication”[tiab] OR “inappropriate medications”[tiab] OR “inappropriate prescription”[tiab] OR “inappropriate prescriptions”[tiab] OR “potentially harmful medication”[tiab] OR “potentially harmful

medications”[tiab] OR “potentially harmful prescribing”[tiab] OR “unsafe medication”[tiab] OR “unsafe medications”[tiab] OR “deprescribing”[tiab]

#3 “medication omission”[tiab] OR “medication omissions”[tiab] OR “prescribing omission”[tiab] OR “prescribing omissions”[tiab] OR “drug

omission”[tiab] OR “drug omissions”[tiab] OR “potential prescribing omission”[tiab] OR “potential prescribing omissions”[tiab] OR “PPO”[tiab] OR “PPOs”[tiab] OR “undertreatment”[tiab] OR “under-treatment”[tiab] OR “underprescribing”[tiab] OR “under-prescribing”[tiab]

#4 “palliative care”[MeSH Terms] OR “palliative care”[tiab] OR “hospice care”[MeSH Terms] OR “hospice”[tiab] OR “hospices”[tiab] OR “terminal

care”[MeSH Terms] OR “terminal care”[tiab] OR “end of life”[tiab] OR “end-of-life”[tiab] OR “life limiting”[tiab] OR “life-limiting”[tiab] OR “terminally ill”[MeSH Terms] OR “terminally ill”[tiab] OR “limited life expectancy”[tiab] OR “limited prognosis”[tiab] OR “terminal illness”[tiab] OR “terminal

stage”[tiab] OR “advanced stage”[tiab] OR “incurable”[tiab] OR “approaching death”[tiab] OR “near death”[tiab] OR “actively dying”[tiab]

#5 “aged”[MeSH Terms] OR “aged, 80 and over”[MeSH Terms] OR “elderly”[tiab] OR “older adult”[tiab] OR “older adults”[tiab] OR “older people”[tiab] OR “older person”[tiab] OR “older persons”[tiab] OR “geriatric”[tiab] OR “geriatrics”[tiab] OR “senior”[tiab] OR “seniors”[tiab] OR “veteran”[tiab] OR “veterans”[tiab]

#6 “prevalence”[MeSH Terms] OR “prevalence”[tiab] OR “epidemiology”[Subheading] OR “utilization”[tiab] OR “utilisation”[tiab] OR “use”[tiab] OR “prescribing”[tiab] OR “prescription”[tiab] OR “prescriptions”[tiab] OR “medication use”[tiab] OR “drug use”[tiab] OR “pattern”[tiab] OR “patterns”[tiab]

#7 #1 OR #2 OR #3

#8 #4 AND #5 AND #6 AND #7

**Database: Embase**

Date searched: April 30, 2026 Limits: Humans, Aged 65+ years

Search Strategy:

#1 ‘polypharmacy’/exp OR ‘polypharmacy’:ab,ti OR ‘medication burden’:ab,ti OR ‘concurrent medication’:ab,ti OR ‘multimedication’:ab,ti OR ‘polymedication’:ab,ti OR ‘multimorbidity’:ab,ti

#2 ‘inappropriate prescribing’/exp OR ‘inappropriate prescribing’:ab,ti OR ‘potentially inappropriate medication’:ab,ti OR ‘potentially inappropriate medications’:ab,ti OR ‘PIM’:ab,ti OR ‘PIMs’:ab,ti OR ‘inappropriate medication’:ab,ti OR ‘inappropriate medications’:ab,ti OR ‘inappropriate

prescription’:ab,ti OR ‘inappropriate prescriptions’:ab,ti OR ‘potentially harmful medication’:ab,ti OR ‘potentially harmful medications’:ab,ti OR

‘potentially harmful prescribing’:ab,ti OR ‘unsafe medication’:ab,ti OR ‘unsafe medications’:ab,ti OR ‘deprescribing’/exp OR ‘deprescribing’:ab,ti

#3 ‘medication omission’:ab,ti OR ‘medication omissions’:ab,ti OR ‘prescribing omission’:ab,ti OR ‘prescribing omissions’:ab,ti OR ‘drug omission’:ab,ti OR ‘drug omissions’:ab,ti OR ‘potential prescribing omission’:ab,ti OR ‘potential prescribing omissions’:ab,ti OR ‘PPO’:ab,ti OR ‘PPOs’:ab,ti OR

‘undertreatment’:ab,ti OR ‘under-treatment’:ab,ti OR ‘underprescribing’:ab,ti OR ‘under-prescribing’:ab,ti

#4 ‘palliative care’/exp OR ‘palliative care’:ab,ti OR ‘hospice care’/exp OR ‘hospice’:ab,ti OR ‘hospices’:ab,ti OR ‘terminal care’/exp OR ‘terminal

care’:ab,ti OR ‘end of life’:ab,ti OR ‘end-of-life’:ab,ti OR ‘life limiting’:ab,ti OR ‘life-limiting’:ab,ti OR ‘terminally ill patient’/exp OR ‘terminally ill’:ab,ti OR ‘limited life expectancy’:ab,ti OR ‘limited prognosis’:ab,ti OR ‘terminal illness’:ab,ti OR ‘terminal stage’:ab,ti OR ‘advanced stage’:ab,ti OR

‘incurable’:ab,ti OR ‘approaching death’:ab,ti OR ‘near death’:ab,ti OR ‘actively dying’:ab,ti

#5 ‘aged’/exp OR ‘elderly’/exp OR ‘elderly’:ab,ti OR ‘older adult’:ab,ti OR ‘older adults’:ab,ti OR ‘older people’:ab,ti OR ‘older person’:ab,ti OR ‘older persons’:ab,ti OR ‘geriatric’:ab,ti OR ‘geriatrics’:ab,ti OR ‘senior’:ab,ti OR ‘seniors’:ab,ti OR ‘veteran’:ab,ti OR ‘veterans’:ab,ti

#6 ‘prevalence’/exp OR ‘prevalence’:ab,ti OR ‘epidemiology’/exp OR ‘utilization’:ab,ti OR ‘utilisation’:ab,ti OR ‘use’:ab,ti OR ‘prescribing’:ab,ti OR ‘prescription’:ab,ti OR ‘prescriptions’:ab,ti OR ‘medication use’:ab,ti OR ‘drug use’:ab,ti OR ‘pattern’:ab,ti OR ‘patterns’:ab,ti

#7 #1 OR #2 OR #3

#8 #4 AND #5 AND #6 AND #7

**Appendix 3 – List of Excluded studies**

| **Reference (Author, Year)** | **Reason for exclusion** |
| --- | --- |
| (Cruikshank et al., 2013) | Editorials, commentaries, letters, case reports, conference abstracts |
| (Ouslander, 2020) |  |
| (O'Mahony & O'Connor M, 2011) |  |
| (Laroche et al., 2017) |  |
| (McDermott et al., 2018) |  |
| (Fernandez Fradejas et al., 2021) |  |
| (Yan et al., 2019) |  |
| (Huang et al., 2020) |  |
| (Martin-Perez et al., 2022) |  |
| (Cruickshank, 2011) |  |
| (Foo et al., 2019) |  |
| (Howard, 2012) |  |
| (Morin, 2022) |  |
| (Mohamed & Ramsdale, 2025) |  |
| (Rocker et al., 2018) |  |
| (Kim, 2025) |  |
| (Roux et al., 2018) |  |
| (Thorpe et al., 2019) |  |
| (Gonzalez-Benitez et al., 2024) |  |
| (Lavan et al., 2016) |  |
| (Ramdass et al., 2017) |  |
| (Leo-To et al., 2018) |  |
| (Wightman et al., 2022) |  |
| (Tjia et al., 2021) |  |
| (Alwidyan et al., 2021) |  |
| (Lindqvist et al., 2013) | Reviews, Delphi studies |
| (Aparicio et al., 2020) |  |
| (Lorenzl, 2014) | Study published in a language other than English, Chinese or Malay |
| (Mozeluk et al., 2024) |  |
| (Chae et al., 2022) | Life expectancy more than 6 months |
| (Morin et al., 2017) |  |
| (Grande et al., 2017) |  |
| (Matthews et al., 2022) |  |
| (Schöttker et al., 2017) |  |
| (Hong et al., 2020) |  |
| (Ramsdale et al., 2022) |  |
| (Chuang et al., 2017) |  |

| (Lavan et al., 2019) |  |
| --- | --- |
| (Letts et al., 2024) |  |
| (Curtin et al., 2020) |  |
| (Anlay et al., 2025) |  |
| (Chuang et al., 2017) |  |
| (Hattori et al., 2023) |  |
| (Umit et al., 2020) |  |
| (Sevilla-Sanchez et al., 2018) |  |
| (Ierodiakonou et al., 2021) |  |
| (Murali et al., 2023) |  |
| (Jiao et al., 2026) |  |
| (Iurlo et al., 2016) | Life expectancy unclear or not indicated |
| (Charlton et al., 2018) |  |
| (Zueger et al., 2019) |  |
| (Kotlinska-Lemieszek et al., 2014) |  |
| (Takahashi et al., 2021) |  |
| (Karuturi et al., 2018) |  |
| (Karuturi et al., 2019) |  |
| (La et al., 2025) |  |
| (L et al., 2024) |  |
| (Tjia et al., 2014) |  |
| (Yue et al., 2024) |  |
| (Keche et al., 2022) |  |
| (O'Neill & Matthews, 2021) |  |
| (Nightingale et al., 2015) |  |
| (Berner-Sharma et al., 2025) |  |
| (Machado et al., 2023) |  |
| (Whalen et al., 2014) |  |
| (Pasina et al., 2020) |  |
| (Vrijkorte et al., 2020) |  |
| (Koh & Koo, 2002) |  |
| (Sera et al., 2014) |  |
| (Stokes et al., 2009) |  |
| (Salvato et al., 2003) |  |
| (Husebo et al., 2021) |  |
| (Leiss et al., 2015) |  |
| (Hoemme et al., 2019) |  |
| (Wastesson et al., 2016) |  |
| (Brecher & West, 2016) |  |
| (Al-Azayzih et al., 2024) |  |

| (Koppel et al., 2018) |  |
| --- | --- |
| (Schelin et al., 2025) |  |
| (White et al., 2019) |  |
| (Niznik et al., 2020) | Not relevant to prevalence and impact of polypharmacy, PIMs or PPOs |
| (Shrestha et al., 2021) |  |
| (Thorpe et al., 2020) |  |
| (Cagle et al., 2021) |  |
| (Saphire et al., 2020) |  |
| (McLean et al., 2013) |  |
| (Segerlantz et al., 2020) | Not all participants aged 65 and older |
| (Peralta et al., 2022) |  |
| (Al-Shahri et al., 2019) |  |
| (Fede et al., 2011) |  |
| (Raijmakers et al., 2013) |  |
| (Resnick & Galik, 2010) |  |
| (Martens et al., 2025) |  |
| (Pommier et al., 2025) |  |
| (Pumsuwan et al., 2025) |  |
| (Wang et al., 2025) |  |
| (Teo et al., 2025) |  |
| (Riveras et al., 2024) |  |
| (Novosadová et al., 2025) |  |
| (Masumoto et al., 2024) |  |
| (Makan et al., 2024) |  |
| (McAdam et al., 2025) |  |
| (Machado et al., 2024) |  |
| (Lindsay et al., 2015) |  |
| (Kiani et al., 2011) |  |
| (Latuga & Levy, 2025) |  |
| (Golčić et al., 2018) |  |
| (Garfinkel et al., 2018) |  |
| (Todd et al., 2014) |  |
| (Gao et al., 2014) |  |
| (Poudel et al., 2019) |  |
| (Moncho et al., 2022) |  |
| (Lundy et al., 2013) |  |
| (Grądalski, 2019) |  |
| (Haider et al., 2021) |  |
| (Shi et al., 2026) |  |
| (Abdelaal et al., 2025) |  |

| (Duncan et al., 2020) |  |
| --- | --- |
| (Murphy et al., 2020) |  |
| (B. J. Russell et al., 2014) |  |
| (Chambers et al., 2026) |  |
| (Currow et al., 2007) |  |
| (Bethany J. Russell et al., 2014) |  |
| (Kawashima et al., 2019) |  |
| (Agar et al., 2009) |  |
| (Paque et al., 2018) |  |
| (van Nordennen et al., 2016) |  |
| (Şenel et al., 2017) |  |
| (Al-Shahri et al., 2020) |  |
| (Muir et al., 2013) |  |
| (Kierner et al., 2016) |  |
| (Drake et al., 2026) |  |
| (Parsons et al., 2015) |  |
| (Hu et al., 2019) |  |
| (Cook et al., 2022) |  |
| (Shega et al., 2015) |  |
| (Frahm et al., 2021) |  |
| (van Merendonk et al., 2022) |  |
| (Frisk et al., 2024) |  |
| (Murphy et al., 2022) |  |
| (Boddaert et al., 2024) |  |
| (Chuang & Mitchell, 2019) |  |
| (Grimm & Barry, 2001) |  |
| (Pak et al., 2016) |  |
| (Poh et al., 2026) |  |
| (Brandt et al., 2005) |  |
| (Ngoc et al., 2022) |  |
| (Fried et al., 2006) |  |
| (Abdul Haseeb et al., 2024) |  |
| (Chess-Williams et al., 2024) |  |
| (Zhang et al., 2026) |  |
| (Zeppetella et al., 1999) |  |
| (Yeh et al., 2022) |  |
| (Wu et al., 2024) |  |
| (Williams et al., 2025) |  |
| (Wilcock et al., 2005) |  |
| (Webber et al., 2024) |  |

| (Tscherny et al., 2025) |  |
| --- | --- |
| (Trapman et al., 2026) |  |
| (Thery et al., 2025) |  |
| (Tan et al., 2024) |  |
| (Szigeti et al., 2024) |  |
| (Sridharan et al., 2024) |  |
| (Sogaard et al., 2025) |  |
| (Shah et al., 2024) |  |
| (Schouten et al., 2024) |  |
| (Rodin et al., 2024) |  |
| (Ramanathan et al., 2024) |  |
| (Pommier et al., 2025) |  |
| (Piamjariyakul et al., 2024) |  |
| (Oliveira et al., 2016) |  |
| (O'Connor et al., 2024) |  |
| (Nauck et al., 2004) |  |
| (Kutner et al., 2015) |  |
| (Kadono et al., 2025) |  |
| (Hickman et al., 2025) |  |
| (Choi et al., 2024) |  |
| (Brandstetter et al., 2025) |  |
| (Battah et al., 2025) |  |
| (Alwafi et al., 2024) |  |
| (Frasca et al., 2020) |  |
| (Rijpstra et al., 2024) |  |
| (Hjermstad et al., 2024) |  |
| (Golla et al., 2025) |  |
| (Li et al., 2024) |  |
| (Alanazi et al., 2024) | Excludes patients at end of life |

## Appendix 4 – Study Findings, Limitations, and Recommendations across Included Articles

**Study Findings, Limitations, and Recommendations across Included Articles**

| **Author, year** | **Study Findings** | **Limitations** |
| --- | --- | --- |
| Alwidyan, 2024 | - Cancer is the most common reason for hospice admission (88·7%) and cause of death (92·5%) - Polypharmacy is prevalent, with excessive polypharmacy observed - Symptomatic medications most prescribed during last 14 days of life - Significant decrease in medications closer to death - More than half received PIMs - Morin et al.'s criteria identified higher prevalence compared to STOPPFrail - Gastrointestinal and cardiovascular medications most common - 64·2% of discontinued medications considered deprescribed - Limited proactive deprescribing observed - Reactive deprescribing is triggered mainly by swallowing problems and EOL prognosis | - **Retrospective Design:** Lack of information and limited access to reasons for medication discontinuation - **Exclusion of 'When Required' Medications:** Potential underestimation of symptomatic medications - **Lack of Access to Pre-Hospice Admission Files:** Underestimation of certain medication categories due to inability to consider duration of use - **Absence of Clinical Data:** Limited availability of clinical data. Potential overestimation of PIMs due to lack of clinical context |
| Anlay, 2025 | - 29·7% of residents had at least one PIM deprescribed in the last 4 months of life. - NSAIDs were the most frequently discontinued PIM (58·4%), while PPIs were the most prescribed (37·6%) and least discontinued (9·5%). - Deprescribing slightly declined from 31·7% to 27·66% (2014–2019), indicating a small but significant negative trend (AQPC −0·47%). - Publication of STOPPFrail and Bruyère deprescribing guidelines (PPIs, antipsychotics) did not significantly affect deprescribing trends. - Interrupted time series and ARIMA analyses showed no observable change after guideline publication. - The decline in deprescribing was more pronounced among residents ≥85 years, with lower educational attainment, and those hospitalised. | - **Data limitations:** Administrative reimbursement data may not reflect actual medication intake or OTC use. Only 15 of 27 STOPPFrail PIM criteria were applicable due to limited clinical information. Deprescribing was defined using dispensing records, which do not capture dose reductions or tapering and may underestimate deprescribing. - **Methodological limitations:** Retrospective decedent cohort design infers LLE from the date of death. Additionally, only four PIMs were included in the guideline impact analysis due to statistical power constraints. - **Temporal limitation:** Data were available only up to 2019, so more recent trends or delayed effects of the guidelines may not have been captured. |
| Chen, 2010 | - Mean no. of medications before death: 7·90 (SD = 3·27) - Upward trend in prescriptions nearing death - Top prescriptions for constipation, pulmonary care, HTN - Significant correlation: heart disease linked to increased medication use - Medications for pulmonary care and HTN increased from admission to death - Decreased pain relief medication use before death | - **Small Sample Size:** Small number of participants - **Limited geographic area:** Focused on one NH in Taiwan - Data is only collected on medication prescriptions, not on administration details or effects - Study did not consider healthcare practitioner's personal values and attitudes toward chronic care |
| Ham, 2022 | - **PIM Utilisation:** 45% of patients received at least one PIM in their last month of life. - **Medication Analysis:** Patients with PIMs received more different medications (10, SD = 5) compared to those without PIMs (3, SD = 4) (P < 0·001). | - **Data Limitations:** Relies on pharmacy-dispensed data; no confirmation of actual patient medication consumption. - **Timing Limitation:** Examines last-month medication use, potentially overlooking when a patient's treatment was labelled palliative, affecting accurate identification of inappropriate medication use. |

|  | - **Prevalence in the Netherlands:** Almost half of older patients with lung cancer in the Netherlands received PIMs in their last month of life. | - **Underestimation of PIMs:** Medications from non-pharmacy sources were not considered, excluding in-patient pharmacy data for certain patients, and overlooking medications dispensed before the last month but continued, resulting in a limited understanding of the issue in the studied population. - **Indication Ambiguity:** Lacks indications for medication prescriptions, challenging the distinction between preventive and symptom relief purposes. - **Population Representation:** The study, centred on older lung cancer patients, assumes generalisation to other cancer types without explicit validation, potentially overlooking variations. The cohort's representation implies similarities in results across comparable cancer types but lacks explicit acknowledgment or exploration of differences. - **Limited Hospitalisation Data:** In-patient pharmacy data during hospitalisation not fully considered, limiting study comprehensiveness. - **Guideline Reliance:** The effectiveness and relevance to the entire population of the OncPal guideline may be subject to scrutiny. |
| --- | --- | --- |
| Hattori, 2025 | - **Prescribing trends:** After initiation of comprehensive home medical care, most preventive and symptomatic medications decreased, except opioids, which increased. Preventive drugs (statins, anticoagulants, antidiabetics, osteoporosis drugs, and antidementia drugs) were frequently deprescribed, reflecting goals of care for patients with limited life expectancy. Symptomatic medications (e.g., analgesics, laxatives, hypnotics) were adjusted based on patient needs, with increased opioid use suggesting improved pain management. - **Specific observations:** Statins showed the greatest reduction, supporting evidence that they are often unnecessary near end-of-life. Anticoagulants and antidiabetics decreased modestly, likely reflecting efforts to balance survival, quality of life, and adverse event risks. Antidementia and osteoporosis drugs were also deprescribed due to limited benefit. Pain management appeared to improve, indicated by increased opioid use and reduced acetaminophen/NSAID prescribing, while hypnotics and acid suppressants remained common for symptom control. - **Contextual comparison:** Findings align with studies in Japan and internationally showing deprescribing of preventive medications while maintaining symptom management. However, opioid prescribing in Japan remains lower than in Western countries, suggesting potential under prescription in East Asia. | - **Population limitation:** Only patients receiving claims for comprehensive home medical care for advanced-stage cancer were included, potentially excluding similar patients not receiving this service. - **Pre-index deprescribing:** Some medications may have been deprescribed before study inclusion, potentially underestimating overall deprescribing. - **Data limitations:** Claims data cannot capture reasons for deprescribing (e.g., adverse events, time-to-benefit, patient preference) and lack clinical details such as cognitive status, individual context, or prescriber awareness of tools like OncPal or STOPPFrail. - **Medication management complexity:** Some medications require tapering, individualised adjustments, or continuation for safety, which claims data may not capture. - **Healthcare process factors:** Influences such as pharmacist involvement, multidisciplinary care, and advance care planning were not captured. |
| Lee, 2025 | - 48·7% of older patients had PIMs, with a mean of 1·7 ± 2·2 PIMs per patient. - Common PIMs: statins, antihypertensives, antidiabetics, and antiplatelets, reflecting ongoing long-term preventive therapy despite LLE. - Highlights misaligned treatment priorities: chronic disease management may overshadow palliative care goals. - Among patients with moderate-severe cancer pain, 45·5% did not receive opioids, indicating significant prescribing omissions. | - Single-centre study in a South Korean home hospice, limiting generalisability. - Retrospective design using medical records, risking missing data, documentation bias, and incomplete clinical context. - Partial application of STOPPFrail criteria: only cardiology, coagulation, and endocrine domains evaluated; other domains excluded, likely underestimating PIM prevalence. - Small sample size (102 patients), limiting statistical power and adjustment for confounders. - Causes of inappropriate prescribing not assessed (e.g., physician decisions, institutional habits, patient or caregiver preferences). |

|  | - Factors contributing to omissions: physician hesitancy, opioid stigma, procedural barriers, and limited prescribing education. - PIM use was associated with higher odds of prescribing omissions, suggesting concurrent overtreatment (preventive meds) and undertreatment (inadequate pain control). - Diabetes was linked to increased opioid omissions, possibly due to prioritisation of chronic disease management. |  |
| --- | --- | --- |
| Mack, 2020 | - 34% of residents with LLI were prescribed statins. - Statin prevalence varied by age and intensity: 65-75 y (44·0%, high intensity 11·1%), >75 y (31·1%, high intensity 5·4%). - Prognosis <6 months: 23% (65-75 y), 12% (>75 y) on statins. - Factors associated with statin use: minority race/ethnicity, >5 medications, atherosclerotic CVD/risk factors. - Over one-third of clinically compromised long-stay NH residents remained on statins. | - **Limited Generalisability:** Focused exclusively on NH residents with LLI. - **Cross-Sectional Design:** Limits establishment of causation and understanding longitudinal medication patterns, potentially missing dynamic changes over time. - **Varying Operational Definitions of LLI:** May influence medication use interpretation based on different criteria. - **Overestimation of Medication Discontinuation:** Residents stopping medications with remaining supply may cause overestimation. - **Data Collection Limitations:** Influencing factors on statin use not in Minimum Data Set (MDS) may impact analysis. |
| Matlow, 2017 | - 45·0% received PIMs in their last week of life. - Common PIMs included antidementia (63·6%) and lipid-lowering agents (47·8%). - Severe cognitive impairment and fewer signs of health instability were associated with PIM use. - Consulting a neurologist or psychiatrist lowered the likelihood of PIM use. - Prescribing PIMs near end of life may cause harm, emphasising the need for active reassessment by physicians. | - Baseline characteristics from administrative databases only - Cross-sectional design hinders individual appropriateness determination - Unable to access patient or decision maker treatment preferences - Difficulty capturing changes in medication administration, especially in advanced dementia - Final MDS assessment not on a specific day, affecting medication quality indicator accuracy |
| Morin, 2019 | - 32·0% of older adults with LLI continued, and 14·0% initiated, drugs of questionable benefit in the last 3 months of life (e.g., statins, calcium supplements, vitamin D, bisphosphonates, antidementia drugs). - Highest proportions observed among those aged 75–84 years, individuals who died from organ failure, and those with multiple chronic conditions. - Substantial number of older individuals with LLI receive drugs of questionable benefit in their final months. | - Prognostic uncertainty may be underestimated, as clinicians might not predict exact death time, introducing potential bias. - The definition of drugs of questionable benefit relies on European expert consensus with limited empirical evidence. - Some patients may have valid palliative indications for drugs deemed inadequate by study criteria. - The study's criteria for assessing drug benefit might not capture actual clinical benefit determined by prescribing physicians. - Alternative tools like STOPPFrail could offer a more precise assessment but require extensive clinical indication data not routinely available. - The Swedish Prescribed Drug Register only includes data from community pharmacies, excluding ~10% of defined daily doses dispensed annually from hospitals or nursing homes. - The study lacks information on whether patients consumed medications as prescribed, potentially leading to inaccuracies in drug utilisation patterns. |
| Murphy, 2022 | - Median medications rose from 5 (IQR 3–7) to 11 on the day of death for older adults with cancer in palliative care. - PIMs, including lipid-lowering drugs and alpha-blockers, were administered in the last week of life. | - **Retrospective Design:** Limits access to real-time clinical decision-making information. - **Lack of Assessment on Long-Term Prescribing:** Data only covers the final seven days of life, rendering certain criteria related to long-term prescribing inapplicable. |

|  | - Prevalence of PIMs varied: STOPPFrail (v1: 17·2%, v2: 19·4%), OncPal (12·8%), PIP-CPC (30%). - Retrospective study limited clinical tool applicability. - Increasing medications significantly increased PIM risk across all assessment tools. - The study noted a rise in medications for older adults with cancer nearing death, with PIM prevalence varying by assessment tool. | - **Single Site Study:** Not representative of the entire older adult population with cancer, as participants were predominantly male. |
| --- | --- | --- |
| Paque, 2018 | - PIM use consistently increased as individuals approached death. - 20% experienced discontinuation of at least one PIM in the last 4 months of life, while 49% had no discontinuation. - Factors associated with discontinuation included hospitalisation before the last 4 months, living in a nursing home, female gender, and higher medication use from 12 to 6 months before death. - Initial PIM use was high, increasing towards death, with only one in five users experiencing discontinuation. | - Study assumes patient compliance with reimbursed dispensed medications without confirming actual consumption. - Administrative data limitations in Belgium (dispensed quarterly) hinders assessment of concomitant use and appropriateness. - Potential overestimation of discontinuation due to unaccounted OTC availability of some PIMs. - Study includes only PIMs not requiring clinical information, likely underestimating overall PIM prevalence. - Lack of access to clinical patient-level data limits adjustment for comorbidities, introducing the risk of residual confounding. - Caution needed in interpreting low discontinuation prevalence in the population aged 75 and older due to potential biases. - Lack of clinical information restricts estimation of treatment risk–benefit ratio for specific medications in specific patients. - Only considers PIMs not needing clinical information, potentially underestimating the true prevalence of PIMs. - Prognostic uncertainty in the elderly population is not accounted for, introducing possible bias in the analysis. |
| Pasina, 2018 | - Mean drug usage decreased from hospice admission to death (9·7 to 8·7). - Symptomatic drugs appropriately administered at both time points. - Significant decrease in patients receiving preventive medications suitable for deprescription (86·8% to 48·6%; P < 0·0001). - Hospice admission associated with a reduction in commonly prescribed preventive medications. - About half of patients continued to receive avoidable medications (e.g., peptic ulcer, GERD, antithrombotics). | - Limited clinical info and ADR reports hinder assessment of polypharmacy risks and prescribing cascades. - Unable to evaluate deprescribing's impact on QoL, mental status, or physical function due to lack of relevant data. - Study confined to a single Italian hospice, potentially limiting generalisability to other hospice settings in Italy or similar contexts. |
| Rausch, 2020 | - Dementia residents had more PIM before death than those without dementia. - Lipid-lowering agents were commonly prescribed in both groups. - Residents with dementia received more new PIM prescriptions. - Excluding anti-dementia meds, new prescriptions were more common in residents without dementia. - Dementia presence and excessive polypharmacy are linked to new PIM prescriptions. | - Dementia diagnoses based on claims data, leading to potential misclassification, especially for those on anti-dementia medication without a formal diagnosis. - Unreported or undiagnosed individuals, particularly with mild symptoms, may have been excluded, resulting in underestimation of dementia cases and questionable medication use. - Sample bias towards NH residents with advanced dementia, limiting generalisability to those with milder forms. - “Never appropriate” medication list focused on advanced dementia, potentially impacting relevance for those at different dementia stages. |

|  | - High prevalence of dementia residents receiving anti-dementia medications warrants attention. | - Study applied the medication list with and without considering anti-dementia drugs in the assessment. - Data from a single German health insurance provider may not fully represent the entire German population due to variations in demographics and morbidity across different insurance funds. |
| --- | --- | --- |
| Roux, 2019 | **Medication Trends:**   - Mean medications: 6·7 (90 days before death), 7·5 (7 days before death), 5·6 (day of death). - 75·2% had an additional essential drug prescription in the last week. - 79·3% had at least one unnecessary drug deprescribed in the last week.   **Drug Classes:**   - Most prescribed: Analgesics (56·4%). - Most deprescribed: Antithrombotic agents (38·2%).   **Palliative Care and Medication Adaptation:**   - Medication therapy aligned with palliative care goals mainly in the last week of life. - Highlighted need for earlier transition to palliative care to prevent unnecessary drug exposure. - Preferential prescription of essential drugs and deprescription of unnecessary drugs in the last 3 months of life. | **Data Limitations**   - Specialised care prescription data may underestimate community prescriptions. - OTC medications were excluded from the analysis.   **Interpretation Challenges**   - Validated drug categories influenced by indication, route, and dosage. - Drug appropriateness varies in individual clinical circumstances affecting categorisation accuracy. - Lack of criteria for deprescribing at study onset and evolving criteria acknowledged. - Challenge in determining optimal deprescribing time with LLE. - Emphasise uncertainty in deprescribing decisions, stressing regular open discussions between clinicians and patients on goals, risks, and benefits. |
| Sakran, 2024 | - Deprescribing prevalence & feasibility: 91% of patients with <6-month life expectancy had ≥1 medication deprescribed; multidisciplinary team accepted 93% of recommendations; overall   ~20% reduction in medication burden.   - Adverse drug events: 15% of patients had previously unrecognised clinically significant adverse drug events identified and addressed through deprescribing. - Most commonly deprescribed medications: Preventive/long-term benefit drugs, including antihypertensives, statins, antiplatelets, and antidiabetics, which have long time-to-benefit. - Life expectancy assessment: Deprescribing more readily accepted for patients with <6-month life expectancy; oncologists estimated prognosis with ~78% accuracy, supporting feasibility of prognosis-based decisions. - Sustainability: 88% of deprescribed medications remained discontinued after discharge, showing good continuity in primary care. - Multidisciplinary intervention effectiveness: Pharmacist-led medication review (~30 min per patient) followed by team discussion was practical, efficient, and effective in managing medication complexity. | - Deprescribing tools known but not systematically applied, reflecting real-world practice. - No evaluation of patient outcomes (quality of life, adverse events, hospitalisations, costs). - Single time-point intervention; deprescribing ideally requires ongoing assessment. - Limited assessment of pharmacist contributions (e.g., dose adjustments for renal/hepatic impairment). - Modest 20% reduction in medication burden, consistent with prior studies. |

| Sourdet, 2018 | - 7·9% NH residents died within 6 months, receiving more therapies if deceased. - EOL residents had fewer drugs but more opioids, less antipsychotics, lipid-lowering drugs, and antihypertensives. - A significant proportion received PIMs. - Despite prescription quality improvement, over half of identified EOL residents still received PIMs. | - Assumption: Residents deceased within 6 months receive palliative care, causes of death unknown. - Ambiguity in defining "end of life" relies on physician and care team responses, challenging with polypathological elderly subjects. - Drug therapy data collected at study start, lacks updates on changes before residents' deaths. - Unaccounted changes during study period may affect accuracy of observed associations. |
| --- | --- | --- |
| Springer, 2020 | - Overall, 27% of veteran NH residents with LLE or advanced dementia discontinued aspirin. - Discontinuation rate was higher in residents with explicit LP documentation at admission (34% vs. 24%). - Urgent need for evidence-based research to inform decisions on aspirin continuation or discontinuation for secondary prevention in this patient population. | **Limited Generalisability**   - Findings may not apply to non-VA nursing homes, especially those with mostly female residents and non-NH settings. - Incidence and predictors of discontinuation may be specific to EOL patients taking aspirin at admission (48% of those with CAD/stroke/TIA).   **Sensitivity to Gap Length**   - Optimal period for identifying aspirin discontinuation with daily medication records is unknown. - Cumulative incidence estimates sensitive to gap length (fell from 27% to 22% with a 30-day gap instead of 14 days). - Use of daily records allows for a shorter gap but introduces uncertainties about optimal duration.   **Data Limitations**   - Lack of pre-Community Living Centre (CLC) admission medication data introduces uncertainties. - Aspirin users may include veterans taking aspirin before admission and new users.   **Deprescribing Intentions**   - Inability to conclusively determine if identified discontinuations represent intentional deprescribing from administrative records. |
| Toscani, 2019 | - 20% orally fed, 13.4% tube fed in the last 7 days before death. - Median of 5 PIMs prescribed. - 57% acknowledged worsening condition, median 4 days before death. - Full palliative care not achieved, possibly due to insufficient acknowledgement of drug appropriateness and lack of implementation by professionals. - Despite some improvements, barriers to full palliative care implementation persist. | - **Retrospective Nature:** Clinical records for data retrieval may introduce biases. - **Underreported Family Involvement:** Limited information on relatives' decision-making participation. - **Data Collection Issues:** Potential for interpretation problems; clinical records may lack full decision context. - **Limited Generalisability:** Findings specific to NHs in northern Italy may not apply to other regions or healthcare systems. |

**Abbreviations: LLI, Life-Limiting Illness; CVD, Cardiovascular; NH, Nursing Home; PIM, Potentially Inappropriate Medications; OTC, Over-The-Counter Medications; LLE, Limited Life Expectancy; LP, Limited Prognosis; VA, Veterans Affairs; EOL, End of Life; CAD, Coronary Artery Disease; TIA, Transient Ischaemic Attack; ADR, Adverse Drug Reactions; QoL, Quality of Life; GERD, Gastro-oesophageal Reflux Disease; MDS, Minimum Data Set; HTN, Hypertension.*

## Appendix 5 – Methodological quality appraisal of included studies

**23**

**Newcastle Ottawa Scale (NOS) for Cohort Studies**

| **COHORT STUDIES** | **Paque et al., 2018** | **Morin et al., 2019** | **Toscani et al., 2019** | **Murphy et al., 2022** | **Rausch et al., 2020** | **Ham et al., 2022** | **Sourdet et al., 2018** | **Roux et al., 2019** | **Springer et al., 2020** | **Chen et al., 2010** | **Pasina et al., 2018** | **Alwidyan et al., 2024** | **Anlay et al., 2025** | **Hattori et al., 2025** | **Sakran et al., 2024** |
| --- | --- | --- | --- | --- | --- | --- | --- | --- | --- | --- | --- | --- | --- | --- | --- |
| **SELECTION** | | | | | | | | | | | | | | | |
| Representativeness of the exposed cohort | ***** | ***** |  | ***** | ***** | ***** |  |  |  |  |  |  | * | * | * |
| Selection of the non-exposed cohort | ***** | ***** | ***** | ***** | ***** | ***** | ***** | ***** | ***** | ***** | ***** | ***** |  |  |  |
| Ascertainment of exposure | ***** | ***** | ***** | ***** | ***** | ***** | ***** | ***** | ***** | ***** | ***** | ***** | ***** | ***** | ***** |
| Demonstration that outcome of interest was not present at start of study | ***** | ***** | ***** | ***** | ***** | ***** | ***** | ***** | ***** | ***** | ***** | ***** | ***** | ***** |  |
| **COMPARABILITY** | | | | | | | | | | | | | | | |
| Comparability of cohorts on the basis of the design or analysis |  | ***** |  |  | ****** | ****** |  |  | ***** |  |  |  |  |  |  |
| **OUTCOME** | | | | | | | | | | | | | | | |
| Assessment of outcome | ***** | ***** | ***** | ***** | ***** | ***** |  | ***** | ***** |  | ***** | ***** | ***** | ***** | ***** |
| Was follow-up long enough for outcomes to occur | ***** | ***** | ***** | ***** | ***** | ***** | ***** | ***** | ***** | ***** | ***** | ***** | ***** | ***** | ***** |
| Adequacy of follow up of cohorts | ***** | ***** | ***** | ***** | ***** | ***** | ***** | ***** | ***** | ***** | ***** | ***** | ***** | ***** |  |
| **TOTAL** | ********* (7)** | ***********  *** (8)** | ********* (6)** | ********* (7)** | *********** (9)** | ***********  **** (9)** | ******* (5)** | ******** (6)** | ********* (7)** | ******* (5)** | ******** (6)** | ******** (6)** | ******** (6)** | ******** (6)** | ****** (4)** |
| **Verdict (Methodological Quality)** | **High** | **High** | **Medium** | **High** | **High** | **High** | **Medium** | **Medium** | **High** | **Medium** | **Medium** | **Medium** | **Medium** | **Medium** | **Medium** |

***High = ≥7 stars Medium = 4-6 stars Poor = <4 stars***

**Joanna Briggs Institute (JBI) Appraisal Checklist for Analytical Cross-Sectional Studies**

| **JBI Appraisal Checklist For Analytical Cross Sectional Studies** | **Matlow et al., 2017** | **Mack et al., 2020** | **Lee et al., 2025** |
| --- | --- | --- | --- |
| Were the criteria for inclusion in the sample clearly defined? | Yes | Yes | Yes |
| Were the study subjects and the setting described in detail? | Yes | Yes | Yes |
| Was the exposure measured in a valid and reliable way? | Yes | Yes | Yes |
| Were objective, standard criteria used for measurement of the condition? | Yes | Yes | Yes |
| Were confounding factors identified? | Yes | Yes | Yes |
| Were strategies to deal with confounding factors stated? | Yes | Yes | No |
| Were the outcomes measured in a valid and reliable way? | Yes | Yes | Yes |
| Was appropriate statistical analysis used? | Yes | Yes | Yes |
| **Verdict (Methodological Quality)** | **High** | **High** | **High** |
| **Overall appraisal** | **Include** | **Include** | **Include** |

##### Appendix 6 – Subgroup & Sensitivity analyses of Polypharmacy Prevalence

**26**

###### Prevalence of polypharmacy vs excessive polypharmacy within the last 6 months of life

**n/N**

4311/7864

232044/424212

26/31

59009/67328

104/106

104/106

59009/67328

232044/424212

4311/7864

26/31

Prevalence of polypharmacy & excessive polypharmacy within the last 6 months of life stratified by region

**n/N**

4311/7864

59009/67328

104/106

232044/424212

26/31

**295494/499541**

Prevalence of polypharmacy & excessive polypharmacy within the last 6 months of life stratified by setting

n/N

232044/424212

26/31

59009/67328

4311/7864

104/106

295494/499541

**29**

### Prevalence of polypharmacy & excessive polypharmacy within the last 6 months of life

**n/N**

232044/424212

4311/7864

59009/67328

**295494/499541**

**(excluding studies with n ≤ 5,000)**

**30**

**Mean number of medications per patient within the last 6 months of life**

|  | **Mean no. of medications within the last 6 months of life (SD)** | | | | |
| --- | --- | --- | --- | --- | --- |
| **Author, year** | **Last 6 m** | **Last 4 m** | **Last 1 m** | **Last week** | **Day of death** |
| Pasina, 2019 | 9·5 (3·6) |  |  |  | 8·4 (3·0) |
| Paque, 2019 | 6·4 (4·9) | 18·9 (11·8) |  |  |  |
| Morin, 2019 |  | 8·9 (4·7) |  |  |  |
| Ham, 2022 |  |  | 6·0 (6·0) |  |  |
| Roux, 2019 |  | 6·7 (3·3) |  | 7·5 (4·1) | 5·6 (3·6) |
| Chen, 2010 | 6·6 (2·9) | 6·8 (2·9) |  |  | 7·9 (3·3) |
| Sourdet, 2018 | 8·3 (3·8) |  |  |  |  |
| Alwidyan, 2024 |  |  | 14·4 (4·4) | 14·2 (4·2) | 10·4 (3·9) |

**Median number of medications per patient within the last 6 months of life**

|  | **Median no. of medications in the last week of life (IQR)** | | | | | | | |
| --- | --- | --- | --- | --- | --- | --- | --- | --- |
| **Author, year** | **Last 7 d** | **Last 6 d** | **Last 5 d** | **Last 4 d** | **Last 3 d** | **Last 2 d** | **1 d before** | **Day of death** |
| Toscani, 2019 | 5 (3-7) |  |  |  |  |  |  |  |
| Murphy, 2023 | 5 (3-7) | 7 (6-9) | 8 (6-11) | 9 (6-13) | 10 (7-13) | 11 (8-13) | 11 (8-14) | 11 (9-15) |
|  | **Median no. of medications in the last 6 months of life (IQR)** | | | | | | | |
|  | **Pre-intervention** | | | **Deprescribing** | | **Post-intervention** | | |
| Sakran, 2025 | 7 (5-10) | | | 1 (1-2) | | 5 (3-8) | | |

**31**

##### Appendix 7 - Publication bias assessments

**32**

4

#### Doi plot and LFK index for the prevalence of polypharmacy

**within the last 6 months of life**

**33**

5

#### Doi plot and LFK index for the prevalence of potentially

**inappropriate medications within the last 6 months of life**

**34**

6

##### Appendix 8–Subgroup & Sensitivity analyses of Potentially Inappropriate Medication (PIM) Prevalence

**35**

**Prevalence of potentially inappropriate medications within the last 6 months of life stratified by time points**

**n/N**

1835/13241

4311/7864

72823/244865

38/78

35280/67328

7296/13844

369/488

417/498

8405/9298

141/155

18666/74368

18693/58415

31577/67328

8024/9298

136/149

3539/7864

61/106

6946/9298

35/180

4184/9298

121/149

52/149

205/488

**36**

# Mean Global PIM Prevalence Across Time Points

**PIM Prevalence (%)**

**Pasina, 2018****Springer, 2020****Paque, 2018****Morin, 2019****Murphy, 2022****Rausch, 2020**

**Ham, 2022	Roux, 2019	Sourdet, 2018	Mack, 2020	Matlow, 2017	Alwidyan, 2024**

**Anlay, 2025****Lee, 2025****Sakran, 2024**

**100**

**90**

**80**

**70**

**60**

**50**

**40**

**30**

**20**

**10**

**0 0	10	20	30	40	50	60	70	80	90	100	110	120	130	140	150	160	170	180	190**

**Days before death**

**Prevalence of potentially inappropriate medications within the last 6 months of life stratified by setting**

**n/N**

38/78

61/106

369/488

35/180

141/155

136/149

1835/13241

35280/67328

7296/13844

417/498

8405/9298

15989/74368

72823/244865

18693/58415

3539/7864

#### Prevalence of potentially inappropriate medications within the last 6 months of life stratified by region

**n/N**

38/78

141/155

35/180

15989/74368

72823/244865

18693/58415

3539/7864

35280/67328

61/106

369/488

417/498

136/149

1835/13241

7296/13844

8405/9298

#### PIM prevalence stratified by general geriatric tools vs. palliative

**specific tools**

**n/N**

369/488

417/498

23/180

31/180

35/180

15989/74368

72823/244865

18693/58415

3539/7864

38/78

35280/67328

61/106

74/106

8405/9298

136/149

**Prevalence of potentially inappropriate medications within the last 6 months of life stratified by different clinical tools**

**n/N**

35280/67328

8405/9298

18693/58415

74/106

23/180

3539/7864

141/155

369/488

417/498

136/149

31/180

15989/74368

72823/244865

35/180

38/78

61/106

**41**

**PIM prevalence stratified by predominantly cancer patients (≥90%) vs.**

**non-cancer/mixed populations (<90%)**

**n/N**

1835/13241

15989/74368

18693/58415

7296/13844

417/498

8405/9298

136/149

35/180

72823/244865

3539/7864

38/78

35280/67328

61/106

369/488

141/155

**165057/490877**

Prevalence of potentially inappropriate medications within the last 6 months of life (excluding smaller populations)

n/N

1835/13241

15989/74368

72823/244865

18693/58415

3539/7864

35280/67328

7296/13844

8405/9298

**163860/489223**

###### Prevalence of potentially inappropriate medications within the last 6 months of life (low risk of bias only)

n/N

1835/13241

35/180

15989/74368

18693/58415

3539/7864

38/78

35280/67328

7296/13844

8405/9298

**91110/244616**

Prevalence of potentially inappropriate medications within the last 6 months of life (omitted Springer et al.)

n/N

1835/13241

35/180

15989/74368

72823/244865

18693/58415

3539/7864

38/78

35280/67328

61/106

369/488

417/498

8405/9298

141/155

136/149

157761/477033

**Appendix 9. Top 10 Drug Classes for Preventive Medications Identified as PIM at each time points (%)**

|  | **Lipid-modifying** | | | | | **PPI** | | | | | **Antithrombotics** | | | | | **Antidementia** | | | | | **ACEi/ARB** | | | | |
| --- | --- | --- | --- | --- | --- | --- | --- | --- | --- | --- | --- | --- | --- | --- | --- | --- | --- | --- | --- | --- | --- | --- | --- | --- | --- |
|  | Last 6 m | Last 4 m | Last 1 m | Last 7 d | Day of death | Last 6 m | Last 4 m | Last 1 m | Last 7 d | Day of death | Last 6 m | Last 4 m | Last 1 m | Last 7 d | Day of death | Last 6 m | Last 4 m | Last 1 m | Last 7 d | Day of death | Last 6 m | Last 4 m | Last 1 m | Last 7 d | Day of death |
| **Pasina, 2018** |  |  |  |  |  | 75.6 |  |  |  | 42.0 | 32.8 |  |  |  | 10.3 |  |  |  |  |  | 10.7 |  |  |  | 1.6 |
| **Springer, 2020** |  |  |  |  |  | 52.7 |  |  |  |  | 51.0 | 27.0 |  |  |  |  |  |  |  |  |  |  |  |  |  |
| **Paque, 2018** | 21.5 | 25.1 |  |  |  | 28.2 | 51.8 |  |  |  |  |  |  |  |  | 1.1 | 1.0 |  |  |  |  |  |  |  |  |
| **Morin, 2019** | 65.2 |  |  |  |  |  |  |  |  |  |  | 1.5 |  |  |  |  | 81.8 |  |  |  |  | 2.3 |  |  |  |
| **Murphy, 2022** | 4.4 |  |  |  |  |  |  |  | 7.8 |  | 2.8 |  |  | 4.5 |  |  |  |  |  |  | 1.0 |  |  | 1.0 |  |
| **Ham, 2022** |  |  | 9.0 |  |  | 32.0 |  | 32.0 |  |  |  |  | 9.0 |  |  |  |  |  |  |  |  |  | 28 |  |  |
| **Roux, 2019** | 18.8 |  |  |  |  | 19.4 | 29.5 |  |  |  |  | 38.2 |  |  |  |  |  |  |  |  |  | 16.8 |  |  |  |
| **Sourdet, 2018** | 9.8 |  |  |  |  |  |  |  |  |  | 34.9 |  |  |  |  | 19.5 |  |  |  |  | 29.1 |  |  |  |  |
| **Mack, 2020** | 13.9 |  |  |  |  |  |  |  |  |  |  |  |  |  |  |  |  |  |  |  |  |  |  |  |  |
| **Matlow, 2017** | 47.8 |  |  | 23.3 |  |  |  |  |  |  | 17.7 |  |  | 9.5 |  | 63.6 |  |  | 31.7 |  |  |  |  |  |  |
| **Anlay, 2025** |  |  |  |  |  |  | 37.6 |  |  |  |  |  |  |  |  |  |  |  |  |  |  |  |  |  |  |
| **Hattori, 2025** | 77.5 |  |  |  |  |  |  |  |  |  | 45.6 |  |  |  |  | 43.4 |  |  |  |  | 62.3 |  |  |  |  |
| **Lee, 2025** | 25.5 |  |  |  |  |  |  |  |  |  | 15.7 |  |  |  |  |  |  |  |  |  | 17.7 |  |  |  |  |
| **Sakran, 2024** |  |  |  |  |  |  |  |  |  |  | 11.9 |  |  |  |  |  |  |  |  |  | 31.1 |  |  |  |  |
| **Mean Prevalence**  **(%)** | **31.6** | **25.1** | **9.0** | **23.3** | **N/A** | **38.7** | **39.6** | **32.0** | **7.8** | **42.0** | **26.6** | **22.2** | **9.0** | **7.0** | **10.3** | **31.9** | **41.4** | **N/A** | **31.7** | **N/A** | **25.3** | **9.6** | **28.0** | **1.0** | **1.6** |
|  | **Osteoporosis** | | | | | **H2 Antagonist** | | | | | **Vitamins & Minerals (e.g. Vit. D, CaCO3)** | | | | | **Oral antidiabetic** | | | | | **5ARI** | | | | |
|  | **Last 6 m** | **Last 4 m** | **Last 1 m** | **Last 7 d** | **Day of death** | **Last 6 m** | **Last 4 m** | **Last 1 m** | **Last 7 d** | **Day of death** | **Last 6 m** | **Last 4 m** | **Last 1 m** | **Last 7 d** | **Day of death** | **Last 6 m** | **Last 4 m** | **Last 1 m** | **Last 7 d** | **Day of death** | **Last 6 m** | **Last 4 m** | **Last 1 m** | **Last 7 d** | **Day of death** |
| **Pasina, 2018** |  |  |  |  |  |  |  |  |  |  | 2.0 |  |  |  | 0.4 | 5.1 |  |  |  | 0.6 | 5.5 |  |  |  | 1.4 |
| **Springer, 2020** |  |  |  |  |  | 11.2 |  |  |  |  |  |  |  |  |  |  |  |  |  |  |  |  |  |  |  |
| **Paque, 2018** | 6.3 | 8.3 |  |  |  | 6.3 | 13.9 |  |  |  | 4.8 | 11.3 |  |  |  |  |  |  |  |  | 1.6 | 4.5 |  |  |  |
| **Morin, 2019** |  | 59.3 |  |  |  |  |  |  |  |  |  | 64.3 |  |  |  |  |  |  |  |  |  |  |  |  |  |
| **Murphy, 2022** |  |  |  | 1.1 |  |  |  |  |  |  |  |  |  | 2.8 |  |  |  |  | 1.7 |  |  |  |  | 1.7 |  |
| **Ham, 2022** |  |  | 2.0 |  |  |  |  |  |  |  |  |  | 3.0 |  |  |  |  | 6.0 |  |  |  |  |  |  |  |
| **Roux, 2019** |  |  |  |  |  |  | 29.5 |  |  |  |  | 18.8 |  |  |  |  |  |  |  |  |  |  |  |  |  |

| **Sourdet, 2018** |  |  |  |  |  |  |  |  |  |  | 31.1 |  |  |  |  |  |  |  |  |  |  |  |  |  |  |
| --- | --- | --- | --- | --- | --- | --- | --- | --- | --- | --- | --- | --- | --- | --- | --- | --- | --- | --- | --- | --- | --- | --- | --- | --- | --- |
| **Hattori, 2025** | 69.5 |  |  |  |  | 68.8 |  |  |  |  | 69.6 |  |  |  |  | 48.5 |  |  |  |  |  |  |  |  |  |
| **Lee, 2025** |  |  |  |  |  |  |  |  |  |  |  |  |  |  |  | 22.6 |  |  |  |  |  |  |  |  |  |
| **Mean Prevalence**  **(%)** | **37.9** | **33.8** | **2.0** | **1.1** | **N/A** | **28.8** | **21.7** | **N/A** | **N/A** | **N/A** | **26.9** | **31.5** | **3.0** | **2.8** | **0.4** | **25.4** | **N/A** | **6.0** | **1.7** | **0.6** | **3.6** | **4.5** | **N/A** | **1.7** | **1.4** |

***Footnote:*** *PPIs and H2 receptor antagonists were classified as preventive based on the rationale used in the majority of primary studies. They were flagged as PIMs when used prophylactically (stress ulcer prevention) without a clear indication. One included study (Hattori et al., 2025) classified PPIs/H2 antagonists as symptomatic medications and is presented in Appendix 10.*

**Mean prevalence of medication classes with preventive intent identified as PIM at last 6 months, last 4 months, last month, last week and day of death, presented in a bar chart.**

*Abbreviations: PPI, Proton pump inhibitor; ACEi/ARB, Angiotensin-converting enzyme inhibitor/Angiotensin receptor blocker; H2 antagonist, Histamine H2-receptor antagonist; 5-ARI, 5-alpha reductase inhibitor*

*Footnote: Data are pooled descriptive averages from studies reporting PIM prevalence at each time point. These are not longitudinal within-study estimates and should not be interpreted as temporal trends.*

**Appendix 10. Top 10 Drug Classes for Symptomatic Medications Identified as PIM at each time points (%)**

|  | **H2 Antagonist** | | | | | **PPI** | | | | | **NSAID** | | | | | **GI antispasmodic** | | | | | **Oral steroid** | | | | |
| --- | --- | --- | --- | --- | --- | --- | --- | --- | --- | --- | --- | --- | --- | --- | --- | --- | --- | --- | --- | --- | --- | --- | --- | --- | --- |
|  | **Last 6 m** | **Last 4 m** | **Last 1 m** | **Last 7 d** | **Day of death** | **Last 6 m** | **Last 4 m** | **Last 1 m** | **Last 7 d** | **Day of death** | **Last 6 m** | **Last 4 m** | **Last 1 m** | **Last 7 d** | **Day of death** | **Last 6 m** | **Last 4 m** | **Last 1 m** | **Last 7 d** | **Day of death** | **Last 6 m** | **Last 4 m** | **Last 1 m** | **Last 7 d** | **Day of death** |
| **Springer, 2020** |  |  |  |  |  |  |  |  |  |  | 3.7 |  |  |  |  |  |  |  |  |  |  |  |  |  |  |
| **Paque, 2018** |  |  |  |  |  |  |  |  |  |  | 6.8 | 15.8 |  |  |  | 4.1 | 26.6 |  |  |  | 9.9 | 29.3 |  |  |  |
| **Roux, 2019** |  |  |  |  |  |  |  |  |  |  |  | 28.2 |  |  |  |  |  |  |  |  |  |  |  |  |  |
| **Anlay, 2025** |  |  |  |  |  |  |  |  |  |  |  | 58.4 |  |  |  |  |  |  |  |  |  |  |  |  |  |
| **Hattori, 2025** | 68.8 |  |  |  |  | 24.5 |  |  |  |  | 47.8 |  |  |  |  |  |  |  |  |  |  |  |  |  |  |
| **Lee, 2025** |  |  |  |  |  |  |  |  |  |  | 8.8 |  |  |  |  |  |  |  |  |  |  |  |  |  |  |
| **Mean Prevalence**  **(%)** | **68.8** | **N/A** | **N/A** | **N/A** | **N/A** | **24.5** | **N/A** | **N/A** | **N/A** | **N/A** | **16.8** | **34.1** | **N/A** | **N/A** | **N/A** | **4.1** | **26.6** | **N/A** | **N/A** | **N/A** | **9.9** | **29.3** | **N/A** | **N/A** | **N/A** |
|  | **Psycholeptic (e.g.**  **Antipsychotics, BZDs)** | | | | | **Beta blockers** | | | | | **Diuretics** | | | | | **Insulin** | | | | | **Psychoanaleptic (e.g.**  **Antidepressants)** | | | | |
|  | **Last 6 m** | **Last 4 m** | **Last 1 m** | **Last 7 d** | **Day of death** | **Last 6 m** | **Last 4 m** | **Last 1 m** | **Last 7 d** | **Day of death** | **Last 6 m** | **Last 4 m** | **Last 1 m** | **Last 7 d** | **Day of death** | **Last 6 m** | **Last 4 m** | **Last 1 m** | **Last 7 d** | **Day of death** | **Last 6 m** | **Last 4 m** | **Last 1 m** | **Last 7 d** | **Day of death** |
| **Paque, 2018** | 14.4 | 31.4 |  |  |  |  |  |  |  |  |  |  |  |  |  |  |  |  |  |  |  |  |  |  |  |
| **Roux, 2019** |  | 30.8 |  |  |  |  |  |  |  |  |  | 22.8 |  |  |  |  |  |  |  |  |  | 23.5 |  |  |  |
| **Pasina, 2018** |  |  |  |  |  | 19.1 |  |  |  | 5.3 | 11.5 |  |  |  | 2.2 | 10.7 |  |  |  | 3.3 |  |  |  |  |  |
| **Mean Prevalence**  **(%)** | **14.4** | **31.1** | **N/A** | **N/A** | **N/A** | **19.1** | **N/A** | **N/A** | **N/A** | **5.3** | **11.5** | **22.8** | **N/A** | **N/A** | **2.2** | **10.7** | **N/A** | **N/A** | **N/A** | **3.3** | **N/A** | **23.5** | **N/A** | **N/A** | **N/A** |

***Footnote:*** *Opioids are not considered as PIMs in end-of-life care. Where analgesics (including opioids) were deprescribed (e.g., Roux et al., 2019), this occurred because pain had resolved or patients were unconscious, not because opioids were deemed inappropriate. Opioid-related issues (underprescribing of laxatives or strong opioids for cancer pain) are reported as potential prescribing omissions (PPO) in Appendix 11.*

**Mean prevalence of medication classes with symptomatic intent identified as PIM at last 6 months, last 4 months, last month, last week and day of death, presented in a bar chart.**

*Abbreviations: NSAID, Non-steroidal Anti-inflammatory Drugs; GI, Gastrointestinal; B-blockers, Beta-blockers*

*Footnote: Data are pooled descriptive averages from studies reporting PIM prevalence at each time point and not longitudinal within-study estimates.*

**Appendix 11. Prevalence of potential prescribing omissions (PPO) within the last 6 months of life**

| **Study** | **Population** | **PPO definition** | **n** | **PPO**  **cases** | **Prevalence (%)** |
| --- | --- | --- | --- | --- | --- |
| Murphy, 2023 | >65 years with cancer at last 7 days of life | PIP-CPC criteria | 180 | 54 | 30·0 |
| Lee, 2025 | >70 years with cancer at last 6 months of life | Absence of strong opioids for moderate-severe cancer pain based on WHO guidelines | 30 | 23 | 76·6 |
